# Effects of geolocators on hatching success, return rates, breeding movements, and change in body mass in 16 species of Arctic-breeding shorebirds

**DOI:** 10.1186/s40462-016-0077-6

**Published:** 2016-04-29

**Authors:** Emily L. Weiser, Richard B. Lanctot, Stephen C. Brown, José A. Alves, Phil F. Battley, Rebecca Bentzen, Joël Bêty, Mary Anne Bishop, Megan Boldenow, Loïc Bollache, Bruce Casler, Maureen Christie, Jonathan T. Coleman, Jesse R. Conklin, Willow B. English, H. River Gates, Olivier Gilg, Marie-Andrée Giroux, Ken Gosbell, Chris Hassell, Jim Helmericks, Andrew Johnson, Borgný Katrínardóttir, Kari Koivula, Eunbi Kwon, Jean-Francois Lamarre, Johannes Lang, David B. Lank, Nicolas Lecomte, Joe Liebezeit, Vanessa Loverti, Laura McKinnon, Clive Minton, David Mizrahi, Erica Nol, Veli-Matti Pakanen, Johanna Perz, Ron Porter, Jennie Rausch, Jeroen Reneerkens, Nelli Rönkä, Sarah Saalfeld, Nathan Senner, Benoît Sittler, Paul A. Smith, Kristine Sowl, Audrey Taylor, David H. Ward, Stephen Yezerinac, Brett K. Sandercock

**Affiliations:** Division of Biology, Kansas State University, Manhattan, KS USA; US Fish and Wildlife Service, Anchorage, AK USA; Manomet Center for Conservation Sciences, Manomet, MA USA; CESAM, Universidade de Aveiro, Campus Universitário de Santiago, Aveiro, Portugal; South Iceland Research Centre, University of Iceland, Selfoss, Iceland; Ecology Group, Institute of Agriculture and Environment, Massey University, Palmerston North, New Zealand; Wildlife Conservation Society, Fairbanks, AK USA; Département de Biologie, Chimie et Géographie and Centre d’Études Nordiques, Université du Québec à Rimouski, Rimouski, QC Canada; Prince William Sound Science Center, Cordova, AK USA; Department of Biology and Wildlife, University of Alaska Fairbanks, Fairbanks, AK USA; Université de Bourgogne Franche-Comté, Dijon, France; Laboratoire Chrono-Environnement UMR CNRS 6249, Besançon, France; Groupe de Recherche en Ecologie Arctique, Francheville, France; Fallon, NV USA; Victorian Wader Study Group, Victoria, Australia; Queensland Wader Study Group, Shailer Park, Queensland Australia; Chair in Global Flyway Ecology, Conservation Ecology Group, Groningen Institute for Evolutionary Life Sciences (GELIFES), University of Groningen, Groningen, The Netherlands; Department of Biological Sciences, Simon Fraser University, Burnaby, BC Canada; ABR, Inc. - Environmental Research and Services, Anchorage, AK USA; Laboratoire Biogéoscience, Université de Bourgogne, Dijon, France; Canada Research Chair in Polar and Boreal Ecology, Université de Moncton, Moncton, NB Canada; Australasian Wader Studies Group, Victoria, Australia; Global Flyway Network, Broome, WA Australia; Helmericks Homestead, Colville Village, AK USA; Cornell Lab of Ornithology, Cornell University, Ithaca, NY USA; Ecology Department, Icelandic Institute of Natural History, Gardabaer, Iceland; Department of Ecology, University of Oulu, Oulu, Finland; Institute of Animal Ecology and Nature Education, Gonterskirchen, Germany; Centre for Wildlife Ecology, Simon Fraser University, Burnaby, BC Canada; Audubon Society of Portland, Portland, OR USA; US Fish and Wildlife Service, Portland, OR USA; Department of Biology, Trent University, Peterborough, ON Canada; Department of Multidisciplinary Studies, York University Glendon Campus, Toronto, ON Canada; New Jersey Audubon Society, Cape May, NJ USA; Delaware Bay Shorebird Project, Ambler, PA USA; Environment Canada, Yellowknife, NT Canada; Arctic Research Centre, Department of Bioscience, Aarhus University, Roskilde, Denmark; University of Montana, Missoula, MT USA; Institut für Landespflege, University of Freiburg, Freiburg, Germany; Environment Canada, Ottawa, ON Canada; Yukon Delta National Wildlife Refuge, US Fish and Wildlife Service, Bethel, AK USA; Department of Geography and Environmental Studies, University of Alaska Anchorage, Anchorage, AK USA; US Geological Survey, Anchorage, AK USA; Surrey, BC Canada

**Keywords:** Breeding success, Geologger, Global location sensor (GLS), Research impacts, Return rates, Tracking methods, Waders

## Abstract

**Background:**

Geolocators are useful for tracking movements of long-distance migrants, but potential negative effects on birds have not been well studied. We tested for effects of geolocators (0.8–2.0 g total, representing 0.1–3.9 % of mean body mass) on 16 species of migratory shorebirds, including five species with 2–4 subspecies each for a total of 23 study taxa. Study species spanned a range of body sizes (26–1091 g) and eight genera, and were tagged at 23 breeding and eight nonbreeding sites. We compared breeding performance and return rates of birds with geolocators to control groups while controlling for potential confounding variables.

**Results:**

We detected negative effects of tags for three small-bodied species. Geolocators reduced annual return rates for two of 23 taxa: by 63 % for semipalmated sandpipers and by 43 % for the *arcticola* subspecies of dunlin. High resighting effort for geolocator birds could have masked additional negative effects. Geolocators were more likely to negatively affect return rates if the total mass of geolocators and color markers was 2.5–5.8 % of body mass than if tags were 0.3–2.3 % of body mass. Carrying a geolocator reduced nest success by 42 % for semipalmated sandpipers and tripled the probability of partial clutch failure in semipalmated and western sandpipers. Geolocators mounted perpendicular to the leg on a flag had stronger negative effects on nest success than geolocators mounted parallel to the leg on a band. However, parallel-band geolocators were more likely to reduce return rates and cause injuries to the leg. No effects of geolocators were found on breeding movements or changes in body mass. Among-site variation in geolocator effect size was high, suggesting that local factors were important.

**Conclusions:**

Negative effects of geolocators occurred only for three of the smallest species in our dataset, but were substantial when present. Future studies could mitigate impacts of tags by reducing protruding parts and minimizing use of additional markers. Investigators could maximize recovery of tags by strategically deploying geolocators on males, previously marked individuals, and successful breeders, though targeting subsets of a population could bias the resulting migratory movement data in some species.

**Electronic supplementary material:**

The online version of this article (doi:10.1186/s40462-016-0077-6) contains supplementary material, which is available to authorized users.

## Background

Delineating connectivity among breeding, stopover, and nonbreeding sites is necessary to fully understand the biology of migratory species and relevant conservation threats [1]. Efforts to describe long-distance movements have historically been limited to recovery data from individually marked birds, but this approach is useful only for species or areas with large numbers of tag recoveries, such as hunted species or large-scale networks of study sites, and is subject to reporting biases [1, 2]. More recently, devices that record location data and can be carried by animals through a full annual cycle are providing valuable new information on animal movements. Satellite tags and GPS loggers are useful for tracking movements anywhere on the globe, but are still too large to be carried by many small-bodied animals [3]. In contrast, geolocators collect light data used to estimate an animal’s geographic location by the timing of sunrise and sunset relative to an internal clock [4] and have recently been miniaturized to a size that can be carried by small birds [5, 6]. Compared to other devices, geolocators have typically provided relatively poor precision (±130–300 km), but location errors are diminishing as analysis techniques improve [7] and may be small relative to the scale of global migratory movements [8, 9]. Geolocators record data worldwide with no tracking effort, but must be physically retrieved for the data to be accessed. The technology is thus particularly useful for tracking long-distance migrants that show site fidelity at some stage of the annual cycle, and identifying the subset of individuals in a population with the strongest site fidelity could further improve tag recovery [10, 11].

Carrying a tracking device can negatively affect animals [12], which is ethically undesirable and could affect movements and thus bias conclusions drawn from the tracking data. In birds, effects of tracking tags may include higher energy expenditure and stress, shorter flight range, and reduced survival or reproductive output [10, 12, 13]. Behavior may also be strongly affected, even when demographic rates are not, which could affect interpretation of movement data obtained from tracking tags [14]. Most studies and permitting agencies follow guidelines that the relative mass of tags should not exceed 3–5 % of body mass [12, 15–17]. However, drag produced by a protruding device may be as detrimental to flight performance as the additional mass [13], so effects of tags may not be predictable based solely on relative mass [18]. Moreover, the 3–5 % guidelines were developed primarily for tags attached by harness to the back of the bird, but other attachment types may have different impacts on balance, drag, and locomotion. Body size, migration distance, habitat use, and foraging method also may influence effects of tags on a particular species [10, 11].

A recent meta-analysis reported an overall negative effect of geolocators on birds, especially for smaller species, aerial foragers, and projects where geolocators were attached to the leg [10]. The focal studies were primarily on seabirds and songbirds, and effects of tags on other groups of birds are not well known. Better data on migratory routes are urgently needed to inform conservation efforts for shorebirds, as nearly half of shorebird populations worldwide have shown long-term declines, including 61 % of populations in North America and 88 % of species that use the East Asian-Australasian Flyway [19–22]. However, concerns have been expressed about applying tags to shorebirds, especially small-bodied and declining species that may show poor resilience to additional energetic costs [23]. Moreover, many species of shorebirds make long migratory flights over water, where stopping is not possible [24–27], and thus may incur particularly high costs from excess weight or drag. Last, leg attachments have been recommended over harnesses for shorebird species that lose and gain a large proportion of their body mass over the course of their annual migration [23, 28]. Tags attached by harnesses have had strong negative effects on return rates of red knots [29] and dunlin [AT & RBL, unpubl.]. Leg-mounted geolocators are more likely to have negative effects than tags attached by harnesses in other taxa [10], but the mechanism for effects of the leg attachment remains unknown, so it is unclear whether shorebirds would also be harmed by tags attached to the leg.

To date, biologists have not reported any negative effects of geolocators on reproduction or return rates for migratory shorebirds [30–38]. However, the small sample sizes and short duration typical of geolocator studies may hinder detection of impacts. A comprehensive assessment of effects of geolocators on a broad range of shorebirds is needed to test for negative effects and identify methods to minimize impacts of geolocators.

Here, we report effects of geolocators on demographic rates of 16 species of Arctic-breeding shorebirds, including five with 2–4 subspecies each, that were captured at 23 breeding and eight nonbreeding sites across the globe. The total of 23 shorebird taxa represents a broad range of body masses (26–1091 g), taxa (eight genera), and migration distances (9–108° latitude). We had four objectives for this study. First, we quantified species-specific effects of geolocators on nest success, partial hatching of clutches, return rate, interannual breeding movement, and change in body mass. We compared birds fitted with geolocators to control birds marked with color bands at the same sites for each taxon in our dataset using hierarchical models that accounted for random effects. Second, we tested for differences between two types of leg-mounted attachments in effects on return rate, nest success, and partial hatching of clutches. Third, we conducted a meta-analysis across the shorebird taxa in our dataset to test whether effects of geolocators on return rates would be more negative for taxa that were smaller or had longer migration distances. Last, we developed general recommendations for deployment of geolocators to minimize impacts on individuals and maximize geolocator retrieval rates in future studies. Our multi-species analysis is one of the most comprehensive tests of tag effects on wildlife and is the first of its kind for shorebirds.

## Methods

Most of the data included in this analysis were collected as part of field studies conducted by the authors. Movement data and tag impacts from have been published for some of these studies [28, 32, 34, 39–42], but the analyses we present here used additional unpublished data on individual covariates. Where possible, we also included data from previously published studies of shorebirds that provided a direct comparison between control and geolocator birds captured at the same site(s) in the same years [33, 35–37, 43]. Published studies included in our meta-analysis reported the number of birds marked and returned for control and geolocator groups by year, but we could not test for effects of individual covariates on return rates for those studies.

### Study species

We tested for effects of geolocators on 16 species of Arctic-breeding shorebirds, including five species with 2–4 subspecies each for a total of 23 study taxa (Table 1). Our dataset was composed of six small-bodied species, including four sandpipers, one phalarope, and one small plover (<100 g); seven medium-sized species, including seven sandpipers, two turnstones, one plover, and a snipe (100–200 g); and three large-bodied species, including a godwit and two curlews (300–1100 g; Table 1).

**Table 1:** Characteristics of Arctic-breeding shorebirds included in our analysis of geolocator effects

Species code	Common name	Scientific name	# sites	# captures		Mean body mass (g)	Mean migration (° latitude)	Geolocator			Max % body mass of markers + geo
				Control	Geo-locator			Attachment type^a^	Total mass (g)^b^	% mean body mass	
SESA	Semipalmated sandpiper	*Calidris pusilla*	7	949	224	26	65^c^	PEF, PAB	0.8–1.0	3.3–3.9	5.8
WESA	Western sandpiper	*C. mauri*	1	276	21	27	49^c^	PAB	1.0	3.7	5.2
RNPH^d^	Red-necked phalarope	*Phalaropus lobatus*	1	21	7	38	66	LLH	1.0	2.6	NA
DUNLsch	*schinzii* dunlin	*C. alpina schinzii*	1	64	30	46	45^d^	PAB	0.8	1.8	2.5
SANDrub	Sanderling	*C. alba rubida*	1	55	44	53	111^e^	PEF	0.8	1.5	
DUNLhud	*hudsonia* dunlin	*C. alpina hudsonia*	1	133	35	57	20^e^	PEF	1.1	1.9	2.9
DUNLpac	*pacifica* dunlin	*C. alpina pacifica*	3	57	124	57	27	PEF	1.1	1.9	2.9
DUNLarc	*arcticola* dunlin	*C. alpina arcticola*	3	255	104	58	30^e^	PEF	1.1	1.9	3.3
SANDalb	Sanderling	*C. alba alba*	3	434	30	59	60	PEF	0.8	1.4	2.3
GSAP	Greater sand plover	*C. leschenaultii*	1	289	59	87^f^	58^e^	PEF	0.9	1.0	1.8
GTTA	Gray-tailed tattler	*Tringa brevipes*	1	160	19	104	88	PAF	1.3	1.3	2.0
RUTUint	*interpres* ruddy turnstone	*Arenaria interpres interpres*	1	112	77	105	113^e^	PEF	0.9	0.9	
RUTUmor	*morinella* ruddy turnstone	*A. i. morinella*	3	62	46	109	70^e^	PEF	0.9	0.8	1.6
BLTU	Black turnstone	*A. melanocephala*	1	51	30	123	9	LLH	2.0	1.6	2.3
REKNrog	*rogersi* red knot	*C. canutus rogersi*	1	11	26	125	108	PAB	1.4	1.1	1.8
REKNruf^g^	*rufa* red knot	*C. c. rufa*	2	711	87	132	100	PAB	1.4–1.7	1.1–1.3	1.6
AMGP	American golden-plover	*Pluvialis dominica*	5	55	129	146	94^c^	PEF	0.9–1.3	0.6–0.9	1.6
GRSN^h^	Great snipe	*Gallinago media*	1	34	45	160	64	PAB	1.3	0.8	NA
GRKN	Great knot	*C. tenuirostris*	1	126	64	195	81^e^	PEF	0.8	0.4	0.8
BTGO	Bar-tailed godwit	*Limosa lapponica*	1	16	58	342	105	PAB	1.8	0.5	1.6
WHIMhud	*hudsonicus* whimbrel	*Numenius phaeopus hudsonicus*	1	31	25	378	54^e^	PAF	1.0	0.3	
WHIMisl	*islandicus* whimbrel	*N. phaeopus islandicus*	1	56	23	438	58^e^	PAF	1.0	0.2	
FECU	Far eastern curlew	*N. madagascariensis*	1	7	23	1091	90^e^	PEF	1.0	0.1	0.3

Species codes follow alpha codes used by the American Ornithologists’ Union. Species and subspecies are sorted by mean body mass. Data are from the authors unless a reference is indicated.*NA* not available

^a^ *PAB* parallel-band, *PEF* perpendicular-flag, *PAF* parallel-flag, *LLH* leg-loop harness (Fig. 2)

^b^Includes attachment materials. Where a range of values is given, multiple models or attachments were used across sites or years

^c^From Thomas et al. [76]

^d^From Smith et al. [37]; total mass of geolocator package is estimated based on our data

^e^Estimated from range maps in Hayman et al. [77] following the methods of Thomas et al. [76]

^f^Published body-mass estimates [78, 79]

^g^From Niles et al. [36] and Burger et al. [33]

^h^From Klaassen et al. [43] and Lindström et al. [35]

### Study sites

**Fig. 1:**
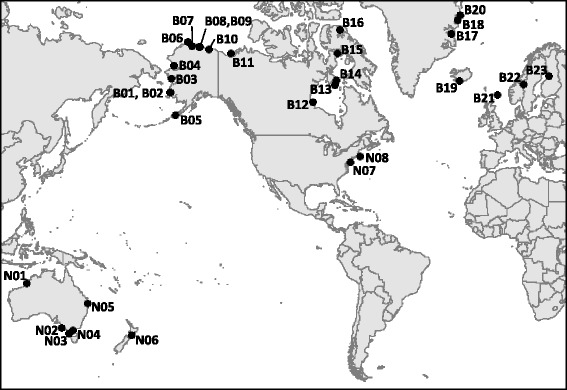
Capture sites included in our analysis of effects of geolocators on Arctic-breeding shorebirds. “*B*” codes indicate breeding sites and “*N*” indicates nonbreeding sites. Map was created with package “ggmap” [80] in R [57]

We marked control birds and deployed geolocators on treatment birds in 2007–2013 at 27 field sites (Additional file 1: Table S1). Previously published studies collected data at four additional sites in 2009–2013, for a total of 31 study sites included in our analysis. We refer to sites by codes based on location: “B” for breeding sites and “N” for nonbreeding sites, with each numbered sequentially from west to east (Fig. 1). At most sites, geolocators were deployed in only a subset of years (Additional file 1: Table S1), with control birds marked concurrently. We also included control birds marked in years when geolocators were not deployed. Including control birds from additional years reduced biases due to seasonal differences in timing of capture which arose when some field crews deployed geolocators on birds captured early in the season until all devices were deployed, after which control birds were marked. For both groups, we included data on resighting and recapturing efforts through the 2014 breeding season and the 2014–2015 nonbreeding season. All sites were included in the analysis of effects of geolocators on return rates, but only breeding sites were used in analyses of effects of tags on components of reproductive performance.

### Capture, marking, and resighting

At breeding sites, we located shorebird nests by rope-dragging or systematically searching study areas, and trapped the attending adults with a bownet or walk-in trap at the nest. At nonbreeding sites, we trapped birds with cannon nets at foraging or roosting locations. Each bird in the control group received a metal band and a unique combination of color markers, while geolocator birds received a geolocator instead of or in addition to color markers. Up to seven color bands (usually 1–4) and one colored flag with or without a field-readable alphanumeric code were applied to each individual, with total mass of markers (metal band, color bands, and flag) ranging from 0.2 to 2.1 % of body mass (Table 1). At some sites, geolocator and control birds were sampled for blood and feathers, and body mass and other morphometrics were recorded. Within each field study, capture and handling methods were consistent between geolocator and control birds, aside from application of the geolocator. All capture, handling, and tagging methods were approved by regulatory committees for animal welfare and permitting agencies for wildlife research. Details for the field methods used by each study are provided in references cited in Additional file 1: Table S1 and Supplementary methods.

**Fig. 2:**
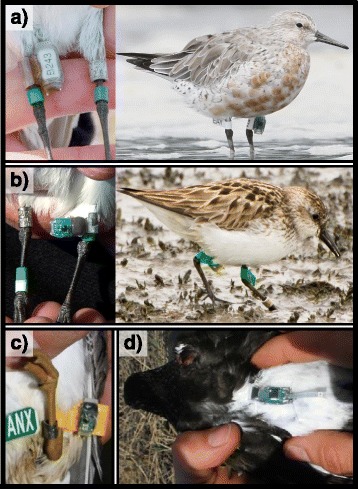
Geolocator attachment methods used in our field studies of Arctic-breeding shorebirds. **a** Mounted on leg band or closely trimmed leg flag, parallel to leg (“parallel-band” attachment), on a semipalmated sandpiper (*left*) and red knot (*right*); (**b**) leg-flag mounted, perpendicular to leg (“perpendicular-flag”), on a sanderling (*left*) and semipalmated sandpiper (*right*); (**c**) leg-flag mounted, parallel to leg (“parallel-flag”) on a gray-tailed tattler; (**d**) mounted on the back with leg-loop harness (“harness”) on a black turnstone. Images are not to scale

Geolocators were usually tied and glued to a plastic leg flag or band for attachment to the shorebirds in our dataset. Leg-mounted geolocators were applied on the tibia; some were oriented parallel to the leg on a flag or band, while others were perpendicular on a flag (Fig. 2). The perpendicular-flag attachment was used after the parallel-band attachment appeared to cause calluses on the lower legs of some birds (see Results). In two studies, geolocators were glued to the rump and secured with an elastic leg-loop harness (see Additional file 1: Supplementary methods for details). Seven models of geolocators were used depending on the site and year: Lotek MK5780 (1.5 g), British Antarctic Survey (BAS) MK14 (1.4 g), BAS MK10B (1.1 g), Biotrack MK5093 (1.1 g), BAS MK20A (0.8 g), Swiss Ornithological Institute SOI-GDL2 v2.3 (0.67 g), and Intigeo W65A9 (0.65 g). The total mass of the geolocator and attachment represented 0.1–3.9 % of body mass depending on taxon, geolocator model, and attachment materials (Table 1).

We included both initial captures of unmarked birds and recaptures of previously marked birds as “capture events.” Each individual could therefore have more than one capture record, and could transition from the control group to the geolocator group if it had a tag applied in a year after the first year of marking. However, once a geolocator bird had its device removed, we did not include the individual in the control group to avoid potential bias from any long-term effects. We also excluded all birds marked as juveniles, because Arctic-breeding shorebirds have low natal philopatry [44] and juveniles marked at nonbreeding sites may delay migration to breeding areas until at least their second year [45].

### Reproduction

#### Nest success

If geolocators are a handicap, we predicted that nest success would be lower for geolocator birds than for control birds. For example, geolocators could present an energetic handicap, increase the probability of parents abandoning a nest, or change nest attendance behavior such that the nest would be more vulnerable to predation [46]. For this analysis, we restricted our sample to a subset of nine breeding sites with both geolocator and control groups and without differences between groups in date or nest age at which birds were captured (*t*-test by species and site, *p* ≥ 0.05). Following capture of a geolocator or control bird, we monitored the nest at which the bird was captured and recorded nest fates as successful, failed, or unknown. We considered a nest to be *successful* if at least one newly hatched chick was observed in the nest, or if eggshells indicative of hatching were found in the nest within 4 days of the expected hatch date [47]. We classified nests as *failed* if all eggs disappeared with no evidence of hatching, and recorded fate as *unknown* if evidence of nest fate was unclear or conflicting, or if the nest was not monitored until the expected hatch date [47]. We excluded nests from the nest fate analysis if nest fate was unknown. A nest was included in the geolocator group if at least one attending parent carried a geolocator; to maximize statistical power, we did not differentiate between nests with one (*N* = 142 nests across all species) versus two (*N* = 64) parents with geolocators.

Based on observations of egg laying or egg flotation data [48], most nests were found early in the incubation period for both groups (geolocator: median = 19 % of incubation period, SD = 24 %; control: median = 14 %, SD = 21 %), across species, sites, and years. We therefore used apparent nest success as our response variable, rather than calculating daily nest survival with known-fate models. Assessing apparent success enabled us to make use of our full dataset, including nests for which we had no estimate of initiation date. For each species, we estimated the effect of geolocators on the binomial response of nest success with a generalized linear mixed model (GLMM) and model averaging (see Statistical methods below). We included site and year as random effects to control for spatial or temporal variation.

#### Cause of failure

For species that showed an effect of geolocators on nest success, we tested for a relationship between geolocators and the cause of failure to investigate a potential mechanism for reduced nest success. We used a GLMM (logit link) and included site and year as random effects. Recorded causes of failure included predation, abandonment, failure to hatch, egg damage, or unknown [47].

#### Partial hatching

For successful nests that hatched at least one egg, we tested for an effect of geolocators on the probability that at least one egg from the clutch would remain unhatched. If geolocators damaged eggs or changed parental incubation behavior, the incidence of unhatched eggs could be higher relative to control nests. Not all sites recorded whether eggs remained unhatched in successful nests, so we used a subset of eight sites for this analysis. We included only nests in which at least one egg was known to have hatched, which further reduced sample sizes. We therefore tested for a fixed effect of geolocators on the presence or absence of unhatched egg(s) as a binomial response in one GLMM with random effects of species, site, and year. If an egg was known to be damaged by research activities other than presence of a geolocator, such as disturbance when the nest was found or when the eggs were measured, we excluded the nest from this analysis.

### Return rates

We recorded which birds returned in the year after capture by resighting or recapturing marked individuals at the capture sites. Effort to retrieve geolocators was typically highest during the year following deployment, so we used the 1-year return rate as a binomial response variable rather than estimating apparent survival and detection probabilities from multi-year datasets [49]. We excluded the few geolocator birds that returned without their geolocator.

To recover geolocators and obtain movement data, resighting or recapturing effort was typically higher for geolocator birds than for control birds. Geolocator birds were more conspicuous than control birds, and thus were likely resighted more frequently, especially at nonbreeding sites. However, resighting effort was not recorded in most of our studies, so we were unable to model observer effort explicitly. Higher search effort for birds with geolocators could mask negative effects or generate apparent positive effects of geolocators on return rates.

#### Species-specific effects on return rates

We expected that return rates would be lower for geolocator birds if carrying the device increased the risk of mortality due to energetic stress or predation, or reduced site fidelity, breeding propensity, or ability to compete for foraging or breeding territories or mates. For five species, body mass and migration route varied among 2–4 subspecies: *pacifica, arcticola, hudsonia,* and *schinzii* dunlin, *rubida* and *alba* sanderlings, *interpres* and *morinella* ruddy turnstones, *rogersi* and *rufa* red knots, and *hudsonicus* and *islandicus* whimbrel (Table 1). We therefore modeled return rates separately for each subspecies. Our sample sizes were lower for other analyses, so we pooled subspecies within each species in all other analyses for dunlin and whimbrel. Only one subspecies of sanderling or ruddy turnstone was captured at breeding sites and included in other analyses, whereas red knots were captured only at nonbreeding sites and were not included in analyses of breeding performance.

For each species or subspecies, we estimated the effect of geolocators on return rate as a binomial response with a GLMM and model averaging (see Statistical methods). We included random effects of site, year, and individual where necessary to control for pseudoreplication. For the analysis of return rates, we had sufficient sample sizes to include additional variables as fixed effects (Table 2), both to control for potential confounding variables and to identify characteristics of individuals that could be targeted to maximize geolocator recovery rates in future studies. We also included an estimate of the approximate total mass of all markers per individual, including color bands, leg flags, and metal bands, to assess whether markers affected return rates in combination with geolocators. Even for tags of the same size and type, mass varied by the material used to construct the tags, and we did not have information on what material was used for every individual. Therefore, we used estimates of marker mass for each band size and tag type to estimate total marker mass per individual.

**Table 2:** Explanatory variables tested for effects on demographic rates of Arctic-breeding shorebirds

Explanatory variable	Type of variable	Response				
		Nest success	Partial hatching of clutches	Return rate	Breeding movements	Change in body mass
Geolocator	Fixed (binomial)	X	X	X	X	X
Sex	Fixed (categorical)	–	–	X	–	–
Nest success^a^	Fixed (categorical)	–	–	X	–	–
Previously marked^b^	Fixed (binomial)	–	–	X	–	–
Body mass	Fixed (continuous)	–	–	X	–	–
Capture date	Fixed (continuous)	–	–	X	–	–
Blood sample	Fixed (binomial)	–	–	X	–	–
Marker mass^c^	Fixed (continuous)	–	–	X	–	–
Difference between capture and recapture date	Fixed (continuous)	–	–	–	–	X
Difference between capture and recapture nest age	Fixed (continuous)	–	–	–	–	X
Site	Random on intercept and slope of geolocator effect	X	X	X	X	X
Year	Random on intercept	X	X	X	X	X
Individual	Random on intercept	–	–	X	–	–
Species	Random on intercept	–	X	–	X	X

Geolocator was the primary variable of interest, but other variables were included (denoted by “X”) in species-specific GLMMs to control for potential confounding factors and identify subsets of the population that could be targeted to improve recovery rates of geolocators in future field studies.

^a^For individuals with multiple nest attempts in 1 year, we used the fate of the final attempt as a potential explanatory variable of return rate

^b^Whether the current capture record is a recapture of an individual that was previously marked at the capture site

^c^Total mass of color markers and metal band applied to each individual, not including the geolocator or attachment

Not all explanatory variables were recorded for every individual, depending on species, site, and year. We included unsexed birds and birds with unknown nest fate, despite the effects of unknown states on return rate being biologically uninterpretable, to maximize sample sizes and thus more precisely estimate the effects of geolocators. After developing the top model set (see Statistical methods below) for the subset of birds with complete data for all variables, we dropped any fixed effects that were not important, added back in the birds that were missing data for the dropped variables, and re-ran the model selection procedure to maximize the final sample size used to estimate the geolocator effects.

#### Cross-species meta-analysis of return rates

We expected that effects of geolocators on return rates would be more negative when geolocators represented a higher percentage of the tagged individual’s body mass. Most geolocators applied in our study were of similar mass (Table 1), so the percent mass of the geolocator was closely related to shorebird body mass. We also predicted that return rates would be more negatively affected by geolocators for long-distance than for short-distance migrants, because we expected that the physiological cost of carrying a geolocator would increase with distance traveled. Percent mass and mean migration distance for each taxon are provided in Table 1. We used meta-analytical techniques (detailed in Statistical Methods) to calculate an overall mean effect of geolocators on return rates and test whether the geolocator effect sizes estimated from the species-specific GLMMs were related to each of these explanatory variables. We did not test for cross-species patterns in geolocator effect on other demographic rates, which used more restricted datasets.

### Sublethal effects

Even when birds successfully reproduce and survive, geolocators and other tags may cause sublethal effects such as higher stress levels or differences in behavior [14, 50]. For birds that returned to our capture sites, we tested for effects of geolocators on two interannual sublethal responses: breeding movement and change in body mass.

#### Breeding movements

We used hand-held GPS units to record nest locations (±5 m) at 11 breeding sites that marked both geolocator and control birds. For each bird that returned in the year following capture, we calculated the distance in meters between nest locations in consecutive years to estimate interannual breeding movements. We tested whether geolocator birds moved farther than control birds, which could result from individuals avoiding the capture site following geolocator deployment or being unable to defend their former territory in the following year while they carried a geolocator.

Sample sizes were limited to birds that had known nest locations in two consecutive years, so to maximize statistical power for our test of geolocator effects, we pooled species in one GLMM with a continuous response and Gaussian errors, and included random effects of species, site, and year. We also included three fixed effects: sex, banding history, and nest fate in the capture year (Table 2), as each of these factors can have strong effects on nest-site fidelity in birds [51–53]. For this analysis, we included only nests located on established study plots in both the capture and return year for both groups of birds. Field crews sometimes made special effort to find nests outside study plots for geolocator birds but not control birds, so excluding off-plot nests constrained recorded movements to the same spatial scale for both groups. Movement estimates were therefore biased low but directly comparable between the two groups.

#### Change in body mass

For each individual that was recaptured and weighed at a breeding site, we calculated the percent change in mass between consecutive capture and recapture years. We did not include nonbreeding sites, where body mass was affected by day of the season when birds prepared for or recovered from long-distance migratory flights. We tested whether individuals carrying geolocators lost body mass relative to control birds, which might be expected if carrying the device was energetically costly or if the birds reduced their body mass to compensate for the mass of the geolocator. Only a fraction of individuals were recaptured, so we pooled species in one GLMM with a continuous response and Gaussian errors, and included random effects of species, site, and year. We included the difference between Julian dates of capture and recapture as a variable to account for seasonal changes in body mass. We likewise included the difference in the age of the nest, as estimated by egg flotation [48], at recapture versus capture to account for changes in body mass over the incubation period [54].

### Leg attachment method

We compared effects of two leg-mounted attachment methods, a parallel-band attachment (Fig. 2a) and a perpendicular-flag attachment (Fig. 2b), on reproduction and return rates. Both leg attachments were used on semipalmated sandpipers at site B03 and on American golden-plovers at site B16. Attachment method was confounded with year at each site, and at site B03, the geolocator groups also differed by the presence of an engraved flag with the perpendicular-flag attachment but not with the parallel-band attachment. All other sites used only one type of geolocator attachment on each species, so no other direct comparisons were possible. Our dataset included only two studies that used harness attachments, so we did not test for differences in effects of harness versus leg-mounted geolocators.

We used a GLMM with a random effect of year to test for an effect of attachment method on nest fate for semipalmated sandpipers, but did not have enough records of nests with known fate for American golden-plovers at site B16 to test for a difference there. We also tested for an effect of attachment method return rate for each species. Nest fate was affected by geolocators in semipalmated sandpipers (see Results) and may affect return rate in shorebirds [55], so we included a fixed effect of nest fate as an explanatory variable in the return rate model. We did not include other variables as sample sizes were small.

### Statistical methods

#### Generalized linear mixed models

We used generalized linear mixed models (GLMMs) to produce standardized estimates of effect sizes of geolocators that were comparable across taxa. The GLMM framework enabled us to combine information across sites and years, each of which may have had a small sample size, while including random effects to control for spatial or temporal variation that may have otherwise confounded geolocator effects. We standardized the scale of explanatory variables by subtracting the mean and dividing by two standard deviations with function “standardize” in package “arm” [56] in R version 3.1.3 [57]. We then used function “glmer” in R package “lme4” [58] to build the full model for each species. Full models are described in Table 2, but not all indicated variables were available for some species.

For each species and response, we tested all possible submodels using function “dredge” in R package “MuMIn” [59]. When multiple submodels were well supported (∆AICc < 2), we used model averaging with function “model.avg” in “MuMIn” to estimate effect sizes for variables in the top model set while accounting for model uncertainty. We used the natural average method of model averaging [60], which has been recommended when one or more variables are of particular interest [61], such as the geolocator effect in our study. We then evaluated the relative importance (RI) of each variable, which was calculated as the sum of the Akaike weights of the top models in which the variable was present divided by the sum of the weights of all top models [60]. When covariates are uncorrelated, as in our analysis, the RI value is a valid metric of variable importance [62, 63] and is not affected by the choice of averaging method [60]. When RI is close to 1.00, the variable is present in most models in the top model set. We used RI ≥ 0.80 to indicate a variable with high relative importance.

If we found group effects on demographic rates, we used the GLMMs to predict the expected rate for each group of interest while controlling for random effects and holding other variables at their means. We provide estimates of expected rates to indicate the biological significance of our results.

#### Meta-analysis

We used meta-analytical techniques to test for cross-species patterns in the effect of geolocators on return rates as estimated by the species-specific GLMMs. Coefficients from GLMMs for effects of geolocators (β_geo_) were estimated while controlling for confounding variables and were directly comparable among shorebird taxa.

First, we estimated an overall mean (*M*) and standard error of the geolocator effect on return rate across all 23 species and subspecies, following Borenstein et al. [64]. We weighted the effect size for each taxon by the number of capture events using the “method of moments” assuming a random effect of taxon on the geolocator effect. We also estimated the proportion of variance among taxa that was real rather than a product of random variation within each group with *I*^*2*^ as a metric of the signal-to-noise ratio.

Second, we tested for effects of 1) percent mass of the geolocator, and 2) mean migration distance, on β_geo_ for each taxon. The magnitudes of the mean and standard error of β_geo_ were correlated, so we transformed each taxon-specific β_geo_ to Fisher’s *z* scale to obtain a *z*_*i*_ value for the *i*^th^ taxon [64, 65]. For each explanatory variable, we calculated the contrast weight, *λ*_*i*,_ for each taxon [65]. We calculated *w*_*i*_ as N_*i*_−3, where N_*i*_ is the number of capture events for taxon *i* [65]. We then calculated the *X*-statistic for each explanatory variable as:

$$ X=\frac{{\displaystyle \sum_{i=1}^n{\lambda}_i{z}_i}}{\sqrt{{\displaystyle \sum_{i=1}^n\frac{{\lambda_i}^2}{w_i}}}} $$

where *n* is the number of taxa. We compared the *X*-value to a standard *Z* distribution to obtain a *p*-value and determine whether percent mass or migration distance explained patterns across taxa in geolocator effects on return rates. We used a Bonferroni correction for the significance level because we were separately testing two explanatory variables, and concluded that either variable was significantly related to the geolocator effect when *p* < 0.025.

If the meta-analysis indicated a relationship between β_geo_ and percent mass of the geolocator or migration distance, we used a weighted least squares linear regression to further assess the nature of the relationship. We used the “lm” function in R and weighted each taxon by the total number of captures. We also calculated where the fitted line intersected zero to identify the threshold for percent mass or migration distance beyond which geolocator effects were likely to be negative.

## Results

Our full dataset included 4935 individuals of 16 species (23 species and subspecies) of shorebirds captured at 31 sites (Additional file 1: Table S2). We captured 7 % of individuals in more than year, for a total of 5308 capture events (1–669 captures per site for each species). We deployed 1328 geolocators at 25 % of capture events (5–77 deployed per site for each species; Additional file 1: Table S2).

### Reproduction

We tested for an effect of geolocators on nest success for 1278 nests of six species at nine breeding sites (Additional file 1: Table S2). Sixteen percent of nests (*N* = 206) were attended by one or two parents with a geolocator.

**Fig. 3:**
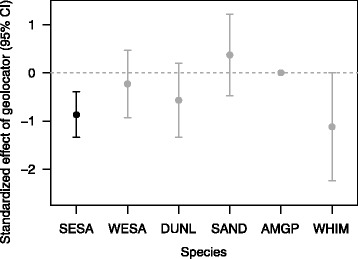
Effect size of geolocator on nest success (mean ± 95 % CI) from species-specific GLMMs. A negative effect indicates that nests attended by birds with geolocators were less likely to hatch than nests of control birds. *Black points* indicate relative importance (RI) of the effect size ≥0.80; *gray points* indicate RI < 0.80 (Additional file 1: Table S3). Models included random effects of site and individual. Species are ordered from smallest to largest; species codes are defined in Table 1 and sample sizes are given in Additional file 1: Table S2

Nest success varied by site and typically was higher for parents without geolocators than if one or both parents carried a geolocator (Additional file 1: Figure S1a). However, the effect of geolocators on nest success had moderate to low RI for all species except semipalmated sandpipers, where RI = 1.00 and the geolocator effect was strongly negative (Fig. 3; Additional file 1: Table S3; top model sets are given in Additional file 1: Table S4). Controlling for random effects and other variables, the GLMM predicted that 77 % of semipalmated sandpipers control nests but only 45 % of geolocator nests were expected to successfully hatch. Eight-four percent of the 168 semipalmated sandpiper nests known to fail were depredated, with abandonment being the next most common cause of failure for both groups (Additional file 1: Figure S2). There was little support for an effect of geolocator on the cause of nest failure (β_geo_ = 0.24, SE = 0.62, RI = 0.27; Additional file 1: Table S5). The effect of geolocators was also strong and negative for whimbrel, but the importance of the effect was ambiguous (RI = 0.56).

We had information on presence or absence of unhatched eggs remaining in the nest cup for successful nests of six species at eight breeding sites (Additional file 1: Table S2). In the two smallest-bodied species, semipalmated and western sandpiper, unhatched eggs were frequently recorded in geolocator nests but rarely in control nests (Additional file 1: Figure S1b). Four larger species showed no evidence of a geolocator effect (Additional file 1: Figure S1b), so we pooled only semipalmated and western sandpipers to test for an effect of geolocators on the probability of unhatched eggs remaining in a hatched nest (*N* = 37 geolocator nests, 304 control nests). The model containing an effect of geolocators was strongly supported (intercept = −1.88; β_geo_ = 1.49, SE = 0.29; *w*_*i*_ = 1.000) over the constant model (∆AICc = 19.36, *w*_*i*_ = 0.000). The top model predicted that unhatched egg(s) were expected to remain in 49 % of successful nests attended by a parent with a geolocator, but only 14 % of successful control nests.

### Return rates

Across our full dataset of capture events, 41 % of control birds and 43 % of birds with geolocators were detected as having returned in the year following capture. Return rates ranged from 4 to 89 % by species and were typically higher for larger-bodied species than for smaller-bodied species, but also varied by site (Additional file 1: Figure S3).

**Fig. 4:**
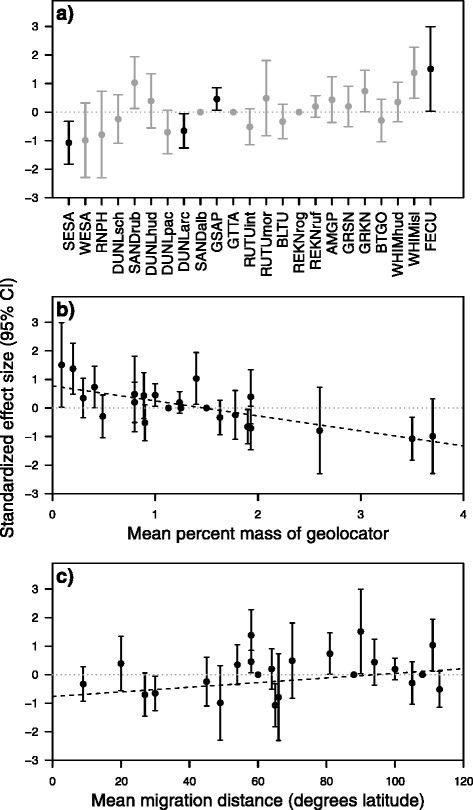
Effects of geolocators on return rates of 23 species and subspecies of Arctic-breeding shorebirds. Values are taxon-specific standardized effect sizes (mean ± 95 % CI) estimated from GLMMs that included random effects of site and individual. Negative values indicate that return rates were reduced for birds with geolocators relative to control birds. A value of zero with no error bars indicates that the geolocator effect was not in the final model for a given species. **a** Effect of geolocator for each taxon, ordered by body mass (smallest to largest). *Black points* indicate relative importance (RI) of the geolocator effect ≥0.80 and *gray points* indicate RI < 0.80. Species codes are defined in Table 1 and sample sizes are given in Additional file 1: Table S2. **b** Relationship between the geolocator effect and the percent of mean body mass represented by the geolocator for each species or subspecies. **c** Relationship between the geolocator effect and mean migration distance for each taxon. The *dashed lines* in (**b**) and (**c**) are fitted lines from weighted least squares linear regression

Presence of a geolocator was not an important predictor of return rates for 19 of 23 taxa of shorebirds (RI < 0.80; Table 3). Geolocators had a negative effect with high RI for semipalmated sandpipers (RI = 1.00) and the *arcticola* subspecies of dunlin (RI = 0.82; Table 3; Fig. 4a). Five of the six sites with semipalmated sandpipers had lower return rates for geolocator birds than for control birds (Additional file 1: Figure S3), and the averaged model predicted return rates of 35 % for the control group versus 13 % for the geolocator group. Two of the three sites with *arcticola* dunlin had lower return rates for geolocator birds than for control birds (Additional file 1: Figure S3), and the averaged model predicted return rates of 37 % for the control group versus 21 % for the geolocator group in *arcticola* dunlin. In contrast, we found positive effects of geolocators with RI > 0.80 in two species, with expected return rates of 69 % for geolocator birds versus 53 % for control birds for greater sand plovers, and 78 versus 29 % for far eastern curlews (Table 3). A positive effect of geolocators approached but did not exceed our RI threshold (0.70 > RI > 0.80) for *rubida* sanderlings and great knots (Table 3). Four other variables that affected return rates (RI > 0.80) were sex, prior site fidelity, nest fate, and day of capture (Table 3). Males showed higher return rates than females in *schinzii* and *arcticola* dunlin. Previously marked birds were more likely to return in *pacifica* dunlin and western sandpipers. Western sandpipers that successfully hatched a nest were more likely to return than those attending nests that failed, and American golden-plovers were more likely to return if captured early in the season.

**Table 3:** Model-averaged effects of explanatory variables on return rates for each species and subspecies of Arctic-breeding shorebirds

Species	Intercept	Geolocator		Nest fate^a^				Sex^b^				Previously marked^c^		Day of capture		Marker mass		Body mass	
				Hatched		Unknown		Male		Unsexed									
	Mean (SE)	Mean (SE)	RI^d^	Mean (SE)	RI	Mean (SE)	RI	Mean (SE)	RI	Mean (SE)	RI	Mean (SE)	RI	Mean (SE)	RI	Mean (SE)	RI	Mean (SE)	RI
SESA	−0.66 (0.24)	**−1.10 (0.45)**	**1.00**	0.28 (0.15)	0.43	0.15 (0.20)	0.43	0	0	0	0	0.22 (0.14)	0.59	0.05 (0.11)	0.19	−0.34 (0.24)	0.49	0	0
WESA	−0.80 (0.22)	−0.99 (0.67)	0.52	**0.43 (0.22)**	**1.00**	**−0.23 (0.33)**	**1.00**	0.11 (0.19)	0.14	0.60 (0.32)	0.14	**0.46 (0.20)**	**0.91**	−0.12 (0.19)	0.10	−0.52 (0.32)	0.54	0	0
RNPH	−0.41 (0.30)	−0.79 (0.77)	0.37																
DUNLsch	0.95 (0.60)	−0.24 (0.44)	0.28					**0.97 (0.36)**	**1.00**			0	0					0	0
SANDrub	−1.84 (0.45)	1.03 (0.46)	0.72																
DUNLhud	−0.62 (0.16)	0.39 (0.48)	0.22	0	0	0	0					0.15 (0.31)	0.16	−0.25 (0.25)	0.23				
DUNLpac	−0.11 (0.44)	−0.70 (0.39)	0.60	0.33 (0.45)	0.36	−0.34 (0.55)	0.36					**0.71 (0.32)**	**0.88**	−0.47 (0.30)	0.55				
DUNLarc	−0.77 (0.17)	**−0.66 (0.31)**	**0.82**	0	0	0	0	**0.57 (0.19)**	**1.00**	**−0.22 (0.29)**	**1.00**	0.21 (0.19)	0.36	0	0	0	0	0	0
SANDalb	−1.31 (0.24)	0	0									0.32 (0.27)	0.32			0.22 (0.22)	0.22		
GSAP	−0.29 (0.11)	**0.46 (0.20)**	**1.00**																
GTTA	−0.08 (0.10)	0	0									0	0	0.23 (0.20)	0.41			0	0
RUTUint	−1.17 (0.20)	−0.52 (0.32)	0.58																
RUTUmor	−1.11 (0.77)	0.49 (0.67)	0.35					0	0	0	0	0	0					0	0
BLTU	−0.38 (0.61)	−0.33 (0.31)	0.11					0.89 (0.52)	0.55	1.27 (0.75)	0.55			−0.76 (0.75)	0.35	−0.25 (0.30)	0.09	−0.66 (0.34)	0.72
REKNrog	0.26 (0.21)	0	0									0	0					0.79 (0.47)	0.61
REKNruf	−1.00 (0.07)	0.20 (0.19)	0.37																
AMGP	−1.47 (0.47)	0.44 (0.41)	0.20	0.83 (0.61)	0.24	1.01 (0.57)	0.24	0	0	0	0	−0.50 (0.54)	0.11	**−0.97 (0.33)**	**1.00**	−0.75 (0.48)	0.75	−0.55 (0.31)	0.74
GRSN	−0.73 (0.28)	0.20 (0.36)	0.28																
GRKN	0.19 (0.32)	0.74 (0.37)	0.73																
BTGO	0.88 (0.25)	−0.29 (0.38)	0.32					0	0	0	0			0	0	0	0		
WHIMhud	−0.09 (0.24)	0.35 (0.35)	0.24	0	0	0	0	0.43 (0.36)	0.31										
WHIMisl	−0.88 (0.54)	1.38 (0.46)	0.67	0	0	0	0	0.49 (0.38)	0.43										
FECU	−1.09 (0.71)	**1.51 (0.76)**	**1.00**																

Models included random effects of site on both the intercept and the geolocator effect, and a random effect of individual when relevant. Blank indicates a variable not tested for a given species (data unavailable); zero indicates a variable tested but not present in the final model. Species are sorted by ascending body mass, and species codes are defined in Table 1. Explanatory variables are defined in Table 2. Sample sizes are given in Table 2; top model sets are given in Additional file 1: Table S6.

^a^Failed nest was the baseline

^b^Female was the baseline sex

^c^Unmarked was baseline relative to previously marked; failed nest was baseline for nest fate

^d^Relative importance of the variable in the averaged model; bold text indicates RI ≥ 0.80

Our meta-analysis indicated that across the 23 species and subspecies, the mean effect of geolocators on return rate was not different from zero (*M* = −0.07, SE = 0.22), and most variance was attributed to real among-taxon variance rather than statistical noise (*I*^*2*^ *=* 99.7 %). The geolocator effect on return rate was more likely to be negative when the geolocator was a higher percentage of mean body mass (Z = 26.6, *p* < 0.001; Fig. 4b), with the fitted line intersecting zero at 1.5 % (intercept = 0.78, SE_intercept_ = 0.14; β_prop_mass_ = −0.53, SE_prop_mass_ = 0.06; *p* < 0.001; Fig. 4b). Unexpectedly, geolocator effects were also more likely to be negative for taxa with a shorter mean migration distance than for taxa that migrated longer distances (Z = 8.7, *p* < 0.001; Fig. 4c). Linear regression indicated that geolocator effects were likely to be negative when mean migration distance was less than 95° latitude (intercept = −0.77, SE_intercept_ = 0.39; β_distance_ = 0.01, SE_distance_ = 0.01, *p* = 0.14; Fig. 4c).

### Sublethal effects

We documented interannual breeding movements for 634 control birds and 83 geolocator birds with known nest locations in two consecutive years, including five species at 10 sites (Additional file 1: Table S2). Most birds (96 %) moved <500 m between years, with seven individuals (0.01 %) moving >1 km to a maximum of 5.7 km (median = 68 m, SD = 335 m), though it is likely that we failed to detect long-distance movements outside our study plots. Geolocators did not affect breeding movements (Table 4). Instead, movements were strongly affected by sex and nest fate, with the distance moved nearly double for females versus males, or when a nest failed versus hatched (Table 4).

**Table 4:** Effects of explanatory variables on sublethal response variables

Explanatory variable	Interannual breeding movements^a^		Proportional change in body mass	
	Mean (SE)	RI	Mean (SE)	RI
Intercept^b^	4.07 (1.26)		0.002 (0.005)	
Geolocator	0	0	−0.003 (0.007)	0.28
Nest fate: hatched	**−0.66 (0.13)**	**1.00**		
Nest fate: unknown	**−0.18 (0.17)**	**1.00**		
Sex: male	**−0.56 (0.10)**	**1.00**		
Sex: unknown	**−0.26 (0.17)**	**1.00**		
Previously marked	−0.07 (0.11)	0.46		
Difference between recapture and capture dates			−0.001 (0.004)	0.17
Difference between recapture and capture nest ages			0.000 (0.003)	0.15

Values are from the final averaged models for five shorebird species at seven sites pooled. Models included random effects of species, site, and individual. See Table 3 for definitions of abbreviations and bold emphasis. Sample sizes are given in Additional file 1: Table S2; top model sets are given in Additional file 1: Table S7.

^a^Distances between nests in subsequent years were measured in meters and log-transformed

^b^For movement, the intercept represents control females whose nest failed to hatch. For change in body mass, effects of sex and nest fate were not tested, so the intercept represents the control group

We had information on body mass at capture in consecutive years for 341 control birds and 109 geolocator birds across five species and 10 breeding sites (Additional file 1: Table S2). Across all individuals, the mean change in body mass was +0.1 % (SD = 8.6 %). Geolocators did not affect the proportional change in body mass between years (Table 4).

### Leg attachment method

**Fig. 5:**
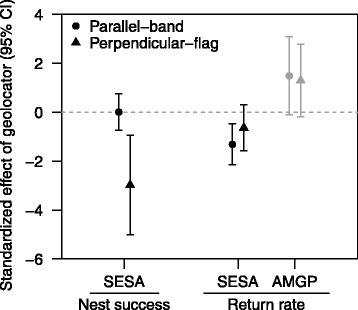
Effect of geolocators with two leg attachment methods on nest success and return rates of two species. Geolocator attachment methods are shown in Fig. 2a. A value of zero indicates no difference from the control group. *Black points* indicate relative importance (RI) of the effect size ≥0.80; *gray points* indicate RI < 0.80 (Additional file 1: Table S8)

At site B03, semipalmated sandpipers with geolocators attached perpendicular to the leg experienced lower nest success (predicted mean = 11 %) than the control group (69 %), whereas nest success of individuals with parallel geolocators (70 %) showed no difference from the control group (Fig. 5; Additional file 1: Table S8). We occasionally observed egg damage, including holes and dents in eggshells, that may have been caused by the perpendicular-flag geolocator attachment at six nests at site B03. All six nests failed, with failure attributed to egg damage for five nests and to predation for the sixth. In contrast, no egg damage was noted at nests of semipalmated sandpipers with parallel-band geolocators. Our field protocols did not specify that eggs should be checked for damage and egg handling was typically avoided to minimize observer effects on the nests, so instances of egg damage at other sites that used the perpendicular-flag attachment may have been overlooked.

The parallel-band attachment but not the perpendicular-flag attachment reduced return rates of semipalmated sandpipers (Fig. 5; Additional file 1: Table S8). Expected return rates were 34 % for control birds but 10 % with parallel-band geolocators, while the expected return rate of 19 % for perpendicular-flag geolocators was not significantly different from the control group. Some birds recaptured with a parallel-band geolocator attachment were noted to have calluses where the end of the geolocator contacted the lower leg as the bird walked (Fig. 2a, left), but the incidence of calluses or other leg injuries was not systematically recorded. Neither attachment method had an important effect on return rate of American golden-plovers (RI = 0.39; Fig. 5; Additional file 1: Table S8).

## Discussion

For most of the 23 shorebird taxa in our analysis, we found no effects of geolocators on demographic rates. However, in contrast to previous studies of single species, our comprehensive analysis for a broad suite of migratory shorebirds found major effects of geolocators on a subset of species. Negative effects of geolocators included reduced nest success, an increased likelihood of partial hatching of clutches, and reduced return rates for three small-bodied species of shorebirds.

### Reproduction

Of six shorebird species for which we had information on nest success, geolocators affected only semipalmated sandpipers, reducing the chance that a nest would hatch by 42 %. For this species, the frequency of each cause of failure was similar between geolocator and control nests that failed to hatch. Some nest failure may have resulted from egg damage, as we also had evidence that geolocators may have damaged eggs: geolocators tripled the probability that unhatched eggs remained in the nest following hatch of the rest of the clutch for semipalmated and western sandpipers. A previous analysis found a similar but nonsignificant trend for unhatched eggs to remain in nests of dunlin with geolocators mounted parallel to the leg on a trimmed flag at site B23 [32]. We conclude that relatively thin eggshells of small-bodied shorebirds may be vulnerable to damage from leg-mounted tags whereas the thicker eggshells of larger species may be more robust. While complete nest failure was attributed to egg damage for only a few nests, additional nests with damaged eggs may have been depredated before the damage was noted by observers. Future geolocator studies should include protocols that balance the need to minimize observer effects on nests with systematically recording egg damage after capture or during incubation, disappearance of eggs during incubation, and presence of unhatched eggs remaining in the nest after hatch of the rest of the clutch.

### Return rates

Geolocators reduced return rates for two small-bodied taxa: semipalmated sandpipers and the *arcticola* subspecies of dunlin. Relative to control birds, individuals were 63 and 43 %, respectively, less likely to return if they carried a geolocator. Lower return rates for geolocator birds could have been caused by higher mortality, permanent emigration, temporary emigration including skipping a breeding season, or a reduced probability of resighting the individual at the capture site in the following year [49]. Limited data from additional years for some species and sites indicate that some geolocator and control birds that were not detected in the year following capture, and thus not included in our return rate estimates, were resighted in a subsequent year. However, we do not have enough data from subsequent years to assess whether geolocator birds may have been more or less likely than control birds to be temporarily absent from the study site. We therefore cannot distinguish among the potential underlying causes of reduced return rates for some geolocator birds in our analysis. However, any lethal or sublethal effects are undesirable impacts on a study species and should be mitigated by reducing geolocator mass and drag, improving attachment methods to avoid egg damage, and minimizing handling time.

Negative effects on return rates were not found for the remaining 20 taxa in our analysis, including one of the smallest species (western sandpipers; Table 1). In two species, we found a positive effect of geolocators on return rate that showed high RI in the model selection process. A positive effect could indicate a true difference between groups, potentially arising from life-history tradeoffs. For example, if geolocators reduce reproductive success, birds that fail to hatch a nest could maintain better body condition and thus experience higher survival over the following nonbreeding season. However, we suspect that the positive effects of geolocators on return rates were likely driven by greater search effort for geolocator than control birds in the return year, as the main goal of each field study was to retrieve movement data from geolocators. Moreover, during resighting efforts at nonbreeding sites, the presence of a device meant that geolocator birds were more conspicuous than flagged control birds in foraging or roosting flocks (see also [36]). Return rates for control birds, but not geolocator birds, therefore could have been underestimated for some species, which might explain the apparent positive effect of geolocators on return rates. The differences in search effort between groups may have masked or reduced some negative consequences of geolocators, so negative effects on return rates may be stronger and more widespread than indicated by our data, though we cannot test this possibility with our dataset.

Across the 23 taxa in our meta-analysis, geolocators were more likely to negatively affect return rates when they represented a higher percentage of body mass. Medium to large taxa (maximum mass of all tags, including geolocators = 0.3–2.3 % of body mass) showed no negative effect of geolocators, while small-bodied taxa (maximum mass of all tags = 2.5–5.8 % of body mass) showed mixed effects. Linear regression indicated that geolocator effects became negative, on average, when mass of the geolocator exceeded 1.5 % of mean body mass, suggesting it would be prudent to further reduce the mass of devices below the 3 % guideline most commonly used. Guidelines developed for backpack-style devices may be too liberal for devices attached to the leg, or effects of tags may be species-specific based on payload and drag in flight [18]. Mass of color bands and flags was not negligible for some species of shorebirds, representing up to 2 % of body mass for the smallest species in our study. Studies that deploy geolocators should avoid adding other markers to birds, especially when mass of geolocators is close to the target threshold for percent body mass. Still, some individuals with total tag mass >5 % of body mass successfully carried geolocators for a year and returned to our study sites, providing movement data to inform conservation decisions [39, 42]. Management and conservation needs should continue to be part of the decision-making process for applying geolocators on small-bodied species of shorebirds, and lower return rates or nest success may be an acceptable tradeoff against new information on migratory movements. However, if tags affect the demographic rates of individuals that carry them, data obtained on migration movements and timing may not be representative of the unmarked population.

In contrast to our prediction but consistent with a previous study [10], our cross-species meta-analysis found that effects of geolocators on return rates were more likely to be negative for taxa with shorter mean migration distances. Larger-bodied shorebirds in our dataset tended to have longer migration distances, so body size may underlie the relationship between migration distance and the effect of geolocators on return rates. Alternatively, contrary to our prediction, physiological stress imposed by carrying a geolocator may not increase with longer migration. Instead, longer-distance migrants may be better able to handle excess mass, possibly because the mass of the geolocator is negligible relative to seasonal fluctuations in body mass [23, 28]. Our estimates of mean migration distance were coarse, as many of the species in our analysis are distributed over a wide range of latitudes in the nonbreeding season. The relationship we found between migration distance and the effect of geolocators on return rates should be retested as more movement data become available to quantify typical migration distances for each species or population.

No negative effects of geolocators on return rates were observed for species captured at nonbreeding sites, but capture region was confounded with body size, with the smallest species in our analysis marked only at breeding sites. The two species in our study that received harness geolocators also showed no negative effects, similar to a previous test of geolocators with this attachment method in a small-bodied shorebird [30]. However, our test of two species is not sufficient to conclude that harness attachments are less detrimental than leg-mounted geolocators for shorebirds. Harness attachments can cause high mortality in shorebirds that migrate longer distances [29; AT & RBL unpubl.] as well as other taxa [17] and should be used with caution for deploying geolocators.

The variation in geolocator effects that we found among species, even shorebirds of similar body size, and among capture sites indicates that factors not measured by our study may be influencing the effects of tags on shorebirds. For example, *arcticola* dunlin winter in East Asia, where coastal habitat is disappearing and declines of shorebirds are unprecedented [21]. In contrast, the other dunlin subspecies in our analysis winter in North America and West Africa, where land use is more stable. For any given species, negative effects of geolocators may be more pronounced if individuals are already affected by habitat degradation or other threats.

### Leg attachment method

When we compared the geolocator attachment methods, we found that semipalmated sandpipers at site B03 with the perpendicular-flag attachment experienced lower nest success than those with the parallel-band attachment or the control group. The difference could have resulted from geolocator orientation, flag versus band attachment, or the combination of a geolocator and a field-readable coded flag, as each of these characteristics differed between groups in this comparison. We suspect eggs may have been damaged by the perpendicular-flag geolocators, though our evidence is circumstantial. In contrast, the parallel-band attachment had no effect on nest success for semipalmated sandpipers, but instead caused calluses and reduced return rates relative to the control group. Use of spacer bands below the geolocator has been recommended to reduce rubbing on the leg [23], but was apparently not fully effective in semipalmated sandpipers. Additional measures such as rounding the corners of leg flags and trimming the contact pins of the geolocator [23, 36] may help minimize damage to tagged birds or their eggs.

### Maximizing recovery of geolocators

Aside from geolocators, four other variables affected return rates, providing new information that could be used to maximize recovery of geolocators or other tags in future field studies. First, males were more likely to return than females for two subspecies of dunlin, likely because males show greater site fidelity in male-territorial species of shorebirds [66–69]. If differential migration of the sexes is not a concern, future studies of male-territorial species could maximize recovery of geolocator data by tagging males. Second, individuals that had been previously banded at the capture site and thus had demonstrated site fidelity were more likely to return in *pacifica* dunlin and western sandpipers. Targeting previously banded birds, when available, for deployment of geolocators may help to maximize tag recovery rates.

Third, relative to individuals whose nest failed, birds that hatched a nest showed higher site fidelity, with higher return rates in western sandpipers and shorter interannual breeding movements for five species pooled. An association between nest success and site fidelity has been shown in previous studies of Arctic-breeding shorebirds as well [55, 70–72]. To maximize tag recovery, geolocators could be applied near the expected hatch date or, if capture of adults attending broods is feasible, after birds have successfully hatched a nest [32]. Either strategy would also minimize egg damage or nest failure from the geolocator. Protection from predation could also improve nest success and thus return rates, either for the general population or for individual nests where geolocators are deployed.

Last, American golden-plovers were more likely to return if captured early in the breeding season, which could be driven by intraseasonal patterns in quality or success of breeders [73]. Future field studies could maximize retrieval of geolocators by selecting individuals with a high probability of returning based on these results. However, any strategy for selecting individuals to carry geolocators has the potential to bias the resulting movement data if timing of movements, duration of stopovers, or areas used may vary with individual characteristics such as sex, age, breeding history, or quality.

## Conclusions

For most species of shorebirds, we found no negative effects of geolocators. However, for some small-bodied taxa (mean body mass ≤58 g), we found negative effects that were substantial. Geolocators reduced nest success, complete hatching of clutches, and return rate for three small-bodied species. Our findings suggest that guidelines for relative mass developed for backpack-style attachments may be too liberal for the leg-mounted tags used in most of our studies. Although we found no effects of geolocators for the majority of species in our analyses, tags may change behavior or physiology of birds even when no effects are observed on demographic rates [14, 50, 74], and long-term impacts may not be predictable from short-term studies [75]. Impacts of geolocators or other leg-mounted tags could be mitigated by minimizing total mass of attached material and by modifying the shape of the tag to reduce damage to eggs or legs. In some species, males, individuals with established site fidelity, and birds that successfully hatched a nest were more likely to return. For species not included in our analysis, a pilot study or previous data from individually marked birds would help strategize deployment of geolocators with the aim of maximizing data recovery, thus reducing the number of birds that need to be tagged and minimizing any potential impacts on the species. Future decisions to deploy devices on small-bodied shorebirds should be made on a case-by-case basis, weighing the potential impacts on individuals and populations against the value of improved knowledge of migratory movements.

## Additional file

Additional file 1: Supplementary methods, Tables and Figures provides further details on field methods, data, and results that support the main text. (PDF 637 kb)
