# Supplementary material for: Effects of geolocators on hatching success, return rates, breeding movements, and change in body mass in 16 species of Arctic-breeding shorebirds
Source: Mov Ecol. 2016 Apr 29;4:12. doi: 10.1186/s40462-016-0077-6 (PMC4850671; doi:10.1186/s40462-016-0077-6)
Supplement: Additional file 1: — Supplementary methods, Tables and Figures provides further details on field methods, data, and results that support the main text. (PDF 637 kb) [file 40462_2016_77_MOESM1_ESM.pdf]

Supplementary Material

Effects of geolocators on hatching success, return rates, breeding movements, and change in body mass in 16 species of Arctic-breeding shorebirds

Weiser et al.
Movement Ecology

Supplementary Methods

The main text provides a general overview of field methods for the authors' studies, with further details or differences described here as needed. Studies are listed in alphabetical order. Sites and species included from each study are listed; see Table 1 for details on species and Table S1 for details on sites.

Published return rates that were included in our analysis were from Lindström et al. [1] and Klaassen et al. [2] (site B22, GRSN), Niles et al. [3] and Burger et al. [4] (sites N07-N08, REKNruf), and Smith et al. [5] (site B21, RNPH).

Species codes used here are defined in Table 1 of the main text; sites are listed in Table S1 and mapped on main Figure 1.

Arctic Shorebird Demographics Network

Sites: B03, B04, B06-B08, B10-B16

Species: SESA, WESA, DUNLpac, DUNLarc, DUNLhud, RUTUmor, AMGP, WHIM

Field methods are detailed by Brown et al. [6].

Battley & Conklin

Site: N06

Species: REKNrog, BTGO

Field methods for this study have been previously published [7–9].

Coleman et al.

Site: N05

Species: GTTA

Birds were caught using mist and cannon nets between March and May, 2011 and between December 2012 and March 2013 for banding and geolocator attachment. Birds developing breeding plumage in those catches were selected for geolocator attachment. Twenty-one geolocators were attached in 2011 and a further 20 in 2012. Geolocators in the first batch were MK10 Bastrak units, supplied by the British Antarctic Survey, with the later batch of Intigeo W65A9RJ devices supplied by Migrate Technology Ltd. Units weighed 1.1 grams and were attached to yellow leg flags using monofilament line and also glued to the flag using a solvent cement. The geolocators were then activated and left in an elevated open space for one week to allow accurate placement and identify any need for recalibration of location data on later

download. The exact date and time of activation for each device was also recorded as a baseline time stamp for the data collected.

All birds caught received a metal bird band issued by the Australian Bird and Bat Banding Society which was placed on the left tarsus along with a green engraved leg flag placed on the right tibia. Those birds selected for geolocator attachment also received a yellow leg flag with the geolocator attached which was placed on the left tibia. Returning birds were caught using cannon nets and the flag with geolocator removed in the field. The metal band and green engraved flag was left on the bird to allow further observations of the individual.

GeoSpoi

Site: B19

Species: WHIMisl

Detailed field methods have been published for the control birds [10, 11]. Geolocators were Intigeo-W65, Migrate Technology Ltd and were attached with a cable tie and leg flag for a total mass of ~ 1 g.

Gilg, Lang, & Bollache

Sites: B17, B20

Species: SANDalb

Field methods followed those described in the main text. Details about field site B17 are available in the literature [12, 13].

Lanctot & Yezerinac

Sites: B02, B05

Species: DUNLpac

Field methods followed those of the Arctic Shorebird Demographics Network [6], except no defined study plots were used and nests were not monitored.

Minton et al.

Sites: N01-N04

Species: SANDrub, GSAP, RUTUint, GRKN, FECU

All control and geolocator birds were caught with cannon nets at high tide roosts on sandy shores at a range of locations. Details have been published for most species [14–16].

Pakanen et al.

Site: B23

Species: DUNLsch

Field methods are detailed in Pakanen et al. [17].

Reneerkens et al.

Site: B18

Species: SANDalb

The field site is approximately 60 km^2 large and was annually visited for 4-8 weeks in June and July by 1-2 teams of 1-3 persons. Researchers, on foot, were solely focusing on sanderlings and used spotting telescopes to read color-banded sanderlings [18, 19]. Adult and juvenile sanderlings were annually color-banded in the whole area. Each year the whole area was searched for nests and color-banded individuals, but especially early in the season snow cover limited the time that can be spend in areas further away from the field station. It is for this reason that we tried as much as possible to deploy geolocators to birds nesting in two sub-areas within the total study area, that were close to the field station and/or bred in areas that are usually snow-free early in the season. Birds were mostly captured on the nest using a small clapnet, but adults guiding chicks were also captured with mistnets. Small spacer bands were applied below the flag with the geolocator to enable free movement of the flag and distribute the weight equally on the leg joint.

Taylor et al.

Site: B01

Species: BLTU

We trimmed feathers from a geolocator-sized area about 2 cm above the uropygial gland and glued the geolocator to this site using Loctite 454 cyanoacrylate instant adhesive. We used 1.0 mm thick Stretch Magic beading cord to secure the geolocator in place with the leg-loop harness described by Warnock & Warnock [10]. The light sensor of the geolocator (Lotek MK 5780, 1.5 g) was on a 20mm stalk attached at 30 degree angle. We searched for all birds that were banded in 2013 when we returned in 2014; this entailed spending ~200 hours searching the plots. We spent about 4 hours off-plot looking specifically for geolocator birds.

Ward & Helmericks

Site: B09

Species: RUTUmor

Observations and captures of ruddy turnstones were made at the Helmericks homestead located on the north end of Anachlik Island at the mouth of the Colville River Delta, Alaska, 2011-2014. All captures were made with a bow trap by baiting birds to a trap site with food and marking followed procedures in Brown et al. [6]. Observations of marked birds were made primarily at the trap site and occasionally at other locations on the island. Handling time averaged 21 + 10 (SD) min (n = 22). Blood and feather samples were taken only on 10 geolocator birds in 2013. No nests were monitored at the Helmericks homestead.

Supplementary References

1. Lindström Å, Alerstam T, Bahlenberg P, Ekblom R, Fox JW, Råghall J, Klaassen RHG: **The migration of the great snipe *Gallinago media*: intriguing variations on a grand theme**. *J Avian Biol* 2015, **46**:1–14.

2. Klaassen RHG, Alerstam T, Carlsson P, Fox JW, Lindström Å: **Great flights by great snipes: long and fast non-stop migration over benign habitats**. *Biol Lett* 2011, **7**:833–835.

3. Niles LJ, Burger J, Porter RR, Dey AD, Minton CDT, Gonzalez PM, Baker AJ, Fox JW, Gordon C: **First results using light level geolocators to track red knots in the Western Hemisphere show rapid and long intercontinental flights and new details of migration pathways**. *Wader Study Gr Bull* 2010, **117**:123–130.

4. Burger J, Niles LJ, Porter RR, Dey AD, Koch S, Gordon C: **Migration and over-wintering of red knots (*Calidris canutus rufa*) along the Atlantic coast of the United States**. *Condor* 2012:302–313.

5. Smith M, Bolton M, Okill DJ, Summers RW, Ellis P, Liechti F, Wilson JD: **Geolocator tagging reveals Pacific migration of red-necked phalarope *Phalaropus lobatus* breeding in Scotland**. *Ibis* 2014, **156**:870–873.

6. Brown SC, Gates HR, Liebezeit JR, Smith PA, Hill BL, Lanctot RB: **Arctic Shorebird Demographics Network Breeding Camp Protocol, Version 5**. U.S. Fish and Wildlife Service and Manomet Center for Conservation Sciences. Available at https://www.aoncadis.org/download/ fileDownload.htm?logicalFileId=722cdc6a-d270-11e4-b276-00c0f03d5b7c; 2014.

7. Conklin JR, Battley PF, Potter MA, Fox JW: **Breeding latitude drives individual schedules in a trans-hemispheric migrant bird.** *Nat Commun* 2010, **1**:67.

8. Conklin JR, Battley PF: **Attachment of geolocators to bar-tailed godwits: a tibia-mounted method with no survival effects or loss of units**. *Wader Study Gr Bull* 2010, **117**:56–58.

9. Conklin JR, Battley PF, Potter MA: **Absolute consistency: individual versus population variation in annual-cycle schedules of a long-distance migrant bird**. *PLoS One* 2013, **8**:e54535.

10. Katrínardóttir B, Alves JA, Sigurjónsdóttir H, Hersteinsson P, Gunnarsson TG: **The effects of habitat type and volcanic eruptions on the breeding demography of Icelandic whimbrels *Numenius phaeopus***. *PLoS One* 2015, **10**:e0131395.

11. Katrínardóttir B, Pálsson S, Gunnarsson TG, Sigurjónsdóttir H: **Sexing Icelandic whimbrels *Numenius phaeopus islandicus* with DNA and biometrics**. *Ringing Migr* 2013, **28**:43–46.

12. Meltofte H, Sittler B, Hansen J: **Breeding performance of tundra birds in high Arctic Northeast Greenland 1988-2007**. *Arct Birds* 2007, **9**:45 – 53.

13. Gilg O, Sittler B, Sabard B, Hurstel A, Sané R, Delattre P, Hanski I: **Functional and numerical responses of four lemming predators in high Arctic Greenland**. *Oikos* 2006, **113**:193–216.

14. Minton CDT, Gosbell K, Johns P, Christie M, Fox JW, Afanasyev V: **Initial results from light level geolocator trials on ruddy turnstone *Arenaria interpres* reveal unexpected migration route**. *Wader Study Gr Bull* 2010, **117**:9–14.

15. Minton C, Gosbell K, Johns P, Christie M, Klaassen M, Hassell C, Boyle A, Jessop R, Fox J: **New insights from geolocators deployed on waders in Australia**. *Wader Study Gr Bull* 2013.

16. Minton C, Gosbell K, Johns P, Christie M, Klaassen M, Hassell C, Boyle A, Jessop R, Fox J: **Geolocator studies on ruddy turnstones *Arenaria interpres* and greater sandplovers *Charadrius***

***leschenaultia* in the East Asian–Australasia Flyway reveal widely different migration strategies**. *Wader Study Gr Bull* 2014, **118**:87–96.

17. Pakanen V, Rönkä N, Thomson RL, Koivula K: **No strong effects of leg-flagged geolocators on return rates or reproduction of a small long-distance migratory shorebird**. *Ornis Fenn* 2015, **92**:101–111.

18. Reneerkens J, van Veelen P, van der Velde M, Luttikhuizen P, Piersma T: **Within-population variation in mating system and parental care patterns in the sanderling (*Calidris alba*) in northeast Greenland**. *Auk* 2014, **131**:235–247.

19. Reneerkens J, Grond K, Schekkerman H, Tulp I, Piersma T: **Do uniparental sanderlings *Calidris alba* increase egg heat input to compensate for low nest attentiveness?** *PLoS One* 2011, **6**:e16834.

20. Pakanen V-M, Luukkonen A, Koivula K: **Nest predation and trampling as management risks in grazed coastal meadows**. *Biodivers Conserv* 2011, **20**:2057–2073.

21. Battley PF, Schuckard R, Melville DS: **Movements of bar-tailed godwits and red knots within New Zealand.** Science for Conservation No. 315. New Zealand Department of Conservation; 2011.

Table S1. Study sites included in the analysis of geolocator effects on shorebirds. Breeding areas are indicated with "B" code while nonbreeding areas are indicated with an "N"; sites in each category are numbered from west to east.

Code	Site name	Study*	Latitude	Longitude	Capture years: Control	Capture years: Geolocator	Species
B01	Tutakoke River, Alaska, USA	1	61.25	-165.62	2013	2013	BLTU
B02	Manokinak River, Alaska, USA	2	61.19	-165.10	-	2010	DUNLpac
B03	Nome, Alaska, USA	3	64.44	-164.96	2010-2013	2011-2013	SESA, WESA
B04	Cape Krusenstern, Alaska, USA	3	67.11	-163.50	2010-2013	2010, 2013	SESA, DUNLpac
B05	Izembek Lagoon, Alaska, USA	2	55.25	-162.85	-	2010	DUNLpac
B06	Barrow, Alaska, USA	3	71.30	-156.76	2010-2013	2010, 2012, 2013	SESA, DUNLarc, AMGP
B07	Ikpikpuk River, Alaska, USA	3	70.55	-154.74	2011-2013	2010, 2013	SESA, DUNLarc, AMGP
B08	Colville River, Alaska, USA	3	70.44	-150.68	2011-2012	2013	RUTUmor
B09	Helmericks Homestead, Alaska, USA	4	70.43	-150.40	2011-2013	2012-2013	RUTUmor
B10	Canning River, Alaska, USA	3	70.12	-145.85	2010-2013	2010, 2013	SESA, DUNLarc
B11	Mackenzie Delta, NWT, Canada	3	69.37	-134.89	2010-2013	2013	SESA
B12	Churchill, Manitoba, Canada	3	58.74	-93.82	2010-2013	2010, 2012, 2013	SESA, DUNLhud, AMGP, WHIMhud
B13	Coats Island, Nunavut, Canada	3	62.86	-82.50	-	2013	SESA
B14	East Bay, Nunavut, Canada	3	63.99	-81.70	2010-2013	2010, 2012, 2013	RUTUmor
B15	Igloolik, Nunavut, Canada	3	69.34	-81.54	-	2013	AMGP, SESA
B16	Bylot Island, Nunavut, Canada	3	73.16	-79.97	2010-2013	2011-2013	AMGP
B17	Karupelv Valley, Greenland	5	72.50	-23.99	2011-2013	2013	SANDalb
B18	Zackenberg, Greenland	6	74.50	-21.00	2007-2013	2011-2013	SANDalb
B19	Keldur, Iceland	7	63.78	-20.12	2009, 2010, 2013	2012-2014	WHIMisl
B20	Hochstetter Forland, Greenland	5	75.15	-19.70	2010-2013	2013	SANDalb
B21	Fetlar RSPB Nature Reserve	8	60.58	-0.78	2012	2012	RNPH
B22	Storulvån, Jämtland, Sweden	9	63.20	12.41	2009, 2011, 2013	2009, 2011, 2013	GRSN
B23	Oulu, Finland	10	64.93	24.87	2002-2013	2013	DUNLsch
N01	Broome, Western Australia	11	-18.00	122.37	2009-2012	2009-2012	GSAP, GRKN
N02	Canunda National Park, South Australia	11	-37.62	140.18	2010	2011	SANDrub
N03	King Island, Tasmania	11	-39.83	143.87	2010-2012	2010-2012	RUTUint
N04	Inverloch, Victoria, Australia	11	-38.62	145.75	2010	2010	FECU
N05	Moreton Bay, Queensland, Australia	12	-27.43	153.17	2011-2012	2012	GTTA
N06	Manawatu River Estuary, Manawatu, NZ	13	-40.47	175.22	2008, 2012	2008, 2012	REKNrog, BTGO
N07	Delaware Bay, New Jersey, USA	14	39.22	-75.02	2009	2009	REKNruf
N08	Monomoy Refuge, Massachusetts, USA	15	41.58	-69.99	2009	2009	REKNruf

* 1) Taylor et al., unpubl.; 2) Lanctot & Yezerinac, unpubl.; 3) Arctic Shorebird Demographics Network, unpubl. [6]; 4) Ward & Helmericks, unpubl.; 5) Gilg, Lang, & Bollache, unpubl.; 6) Reneerkens, unpubl.; 7) GeoSpoi [10, 11]; 8) Smith et al. [5]; 9) Lindström et al. [1, 2]; 10) Pakanen et al. [17, 20]; 11) Minton et al. [14–16]; 12) Coleman, unpubl.; 13) Battley & Conklin [8, 9, 21]; 14) Niles et al. [3]; 15) Burger et al. [4]. See Supplementary Methods for details on unpublished studies and their field methods.

Table S2. Final number of capture events (control; geolocator) used in each analysis for each species-site combination. Species codes are defined in Table 1; site codes are in Table S1.

								Geolocator orientation*	
Species	Site	Nest success	Cause of nest failure	Partial hatching of clutches	Return rate	Breeding movement	Change in body mass	Nest success	Return rate
SESA	B03	86; 43	8; 0	61; 19	197; 60	66; 7	12; 10	71; 43	164; 32; 28
	B04				137; 18	42; 6	15; 7		
	B06	123; 2	60; 11	111; 0	203; 28	96; 2	86; 1		
	B07	73; 20	10; 18	63; 2	158; 29	47; 4	38; 3		
	B10	148; 13	3; 3	86; 1	234; 29	66; 13	9; 13		
	B11	7; 8	24; 23	4; 4	20; 13	3; 1	1; 0		
	B13				0; 35				
	B15				0; 12				
WESA	B03	136; 19	41; 8	90; 11	276; 21	95; 6	51; 6		
RNPH	B21				21; 7				
DUNLsch	B23				57; 30				
SANDrub	N02				55; 44				
DUNLpac	B02				0; 48				
	B04	34; 11	5; 8	26; 10	57; 30	31; 11	14; 11		
	B05				0; 46				
DUNLhud	B12				133; 35	30; 11	4; 16		
DUNLarc	B06	95; 27	8; 1	86; 17	176; 47	124; 6	94; 15		
	B07				52; 35	9; 2	7; 3		
	B10				27; 22	13; 5	1; 4		
SANDalb	B17				47; 7				
	B18	289; 10			284; 15				
	B20				99; 11				
GSAP	N01				289; 59				
GTTA	N05				160; 19				
RUTUint	N03				112; 77				
RUTUmor	B08				9; 5	3; 1			
	B09				44; 22				
	B14				9; 39	0; 1	6; 0		
BLTU	B01				51; 30				
REKNrog	N06				11; 25				
REKNruf	N07				622; 47				
	N08				89; 40				
AMGP	B06	19; 21	0; 7	17; 12	40; 24	6; 3	3; 4		
	B07				0; 5				
	B12	1; 9			2; 14	1; 3			
	B15				0; 15		0; 4		
	B16				13; 71	2; 1	0; 12		13; 47; 24
GRSN	B22				34; 45				
GRKN	N01				126; 64				
BTGO	N06				16; 58				
WHIMhud	B12	25; 21	9; 17	16; 4	31; 24				
WHIMisl	B19	36; 2		18; 1	55; 23				
FECU	N04				7; 23				

* Given as control; PAB geolocator; PAF geolocator (Figure 2).

Table S3. Model-averaged effects of geolocators on nest success for six species of Arctic-breeding shorebirds. Species are sorted by ascending body mass; codes are defined in Table 1. All models included random effects of site and year. Submodel sets are given in Table S4; sample sizes are given in Table 2.

	Intercept	Geolocator	
Species	Mean (SE)	Mean (SE)	RI*
SESA	0.34 (0.28)	**-0.87 (0.24)**	**1.00**
WESA	0.13 (0.32)	-0.23 (0.36)	0.30
DUNL	0.83 (0.17)	-0.57 (0.39)	0.45
SAND	0.51 (0.13)	0.37 (0.43)	0.34
AMGP	0.52 (0.82)	0	0
WHIM	-0.34 (0.29)	-1.12 (0.57)	0.56

* RI = relative importance of the covariate in the averaged model; bold text indicates RI ≥ 0.80.

Table S4. Full model sets for nest success of six species of Arctic-breeding shorebirds. Species codes are defined in Table 1.

Species	Model rank	Intercept (control)	Geolocator[a]	df	Deviance	AICc	ΔAICc	w_i[b]
SESA	1	0.34	X	4	-274.0	556.2	-	0.997
	2	0.21	-	3	-281.0	568.1	11.97	0.003
WESA	1	0.13	-	2	-95.0	194.0	-	0.696
	2	0.15	X	3	-94.8	195.7	1.660	0.304
DUNL	1	0.77	-	3	-65.6	137.4	-	0.547
	2	0.89	X	4	-64.8	137.7	0.38	0.453
SAND	1	0.53	-	2	-149.7	303.3	-	0.658
	2	0.51	X	3	-149.3	304.7	1.310	0.342
AMGP	1	0.52	-	3	-21.5	49.5	-	0.763
	2	0.43	X	4	-21.5	51.8	2.340	0.237
WHIM	1	-0.23	X	4	-53.8	116.2	-	0.563
	2	-0.48	-	3	-55.2	116.7	0.510	0.437

[a] "X" indicates a categorical covariate present in the submodel; "-" indicates a covariate tested in the full model but not present in the submodel.
[b] Submodels are ranked and sorted by descending model weight (w_i).

Table S5. Full model set for the effect of geolocators on cause of nest failure in semipalmated sandpipers. Submodels included random effects of year and site. See Table S4 for definitions of abbreviations.

Model rank	Intercept (control)	Geolocator	df	Deviance	AICc	ΔAICc	w_i
1	2.48	-	3	-45.8	97.6	-	0.726
2	2.40	+	4	-45.7	99.6	1.95	0.274

Table S6. Top model sets for return rate for each species and subspecies of Arctic-breeding shorebirds. Submodels with ΔAICc < 2.0 are shown here, and are sorted by descending model weight (wi) within each species. Covariates were not tested for a given taxon when there was insufficient variation. All submodels included random effects of year, site, and individual if relevant. Species codes are defined in Table 1; see Table S4 for definitions of abbreviations.

Species	Model rank	Inter-cept	Geo-locator	Sex	Nest fate	Previously marked	Body mass	Day of capture	Marker mass	k	Dev-iance	AICc	ΔAICc	w_i
SESA	1	-0.62	X		-	X	-	-	-	7	-747.1	1508.3	-	0.090
	2	-0.75	X		X	X	-	-	-0.37	10	-744.1	1508.3	0.002	0.090
	3	-0.59	X		-	-	-	-	-	6	-748.1	1508.3	0.050	0.088
	4	-0.89	X		X	X	-	-	-	9	-745.1	1508.4	0.066	0.087
	5	-0.55	X		-	X	-	-	-0.32	8	-746.2	1508.4	0.134	0.084
	6	-0.52	X		-	-	-	-	-0.3	7	-747.3	1508.8	0.468	0.071
	7	-0.66	X		X	-	-	-	-0.35	9	-745.4	1509.0	0.707	0.063
	8	-0.76	X		X	-	-	-	-	8	-746.5	1509.1	0.819	0.06
	9	-0.63	X		-	X	-	0.06	-	8	-746.9	1510	1.698	0.039
	10	-0.56	X		-	X	-	0.06	-0.32	9	-746	1510.1	1.810	0.037
	11	-0.6	X		-	-	-	0.05	-	7	-748	1510.2	1.867	0.035
	12	-0.74	X		X	X	-	0.03	-0.37	11	-744	1510.3	1.980	0.034
WESA	1	-0.73	X	-	X	X	-	-	-0.59	7	-195.2	404.8	-	0.120
	2	-0.85	-	-	X	X	-	-	-	5	-197.3	404.9	0.075	0.116
	3	-0.93	-	X	X	X	-	-	-	7	-195.7	405.8	1.006	0.073
	4	-0.86	-	-	X	X	-	-	-0.14	6	-197.0	406.3	1.507	0.057
	5	-0.74	X	-	X	X	-	-0.12	-0.63	8	-195.0	406.5	1.725	0.051
	6	-0.61	X	-	X	-	-	-	-0.67	6	-197.2	406.6	1.854	0.048
	7	-0.83	X	-	X	X	-	-	-	6	-197.2	406.7	1.953	0.045
RNPH	1	-0.47	-							1	-19.3	40.8	-	0.633
	2	-0.30	X							2	-18.7	41.9	1.090	0.367
DUNLsch	1	0.97	-	X		-	-			3	-30.0	66.4	-	0.507
	2	0.90	X	X		-	-			4	-29.9	68.3	1.889	0.197
SANDrub	1	-1.99	X				-			3	-48.5	103.2	-	0.717
	2	-1.46	-				-			2	-50.5	105.1	1.857	0.283
DUNLpac	1	-0.11	X		-	X	-	-		7	-116.6	247.9	-	0.154
	2	0.10	X		-	X	-	-0.41		8	-115.6	248.0	0.047	0.151
	3	-0.23	-		X	X	-	-0.48		9	-114.8	248.6	0.645	0.112
	4	-0.54	-		X	X	-	-		8	-115.9	248.7	0.798	0.103
	5	0.41	X		-	-	-	-0.55		7	-117.2	249.0	1.059	0.091
	6	-0.39	-		-	X	-	-		6	-118.3	249.0	1.088	0.089
	7	-0.08	X		X	X	-	-0.53		10	-114.2	249.7	1.769	0.064
DUNLhud	1	-0.58	-		-	-	-	-		2	-114.7	233.5	-	0.249
	2	-0.58	-		-	-	-	-0.25		3	-114.2	234.5	1.028	0.149
	3	-0.66	X		-	-	-	-		3	-114.3	234.7	1.162	0.139
	4	-0.61	-		-	X	-	-		3	-114.6	235.4	1.838	0.099
DUNLarc	1	-0.76	X	X		-	-			8	-226.7	469.8	-	0.413
	2	-0.78	X	X		X	-			9	-225.9	470.3	0.483	0.324
	3	-0.80	-	X		-	-			7	-228.7	471.7	1.892	0.160
SANDalb	1	-1.32	-			-	-		-	3	-255.9	517.9	-	0.294
	2	-1.33	-			X	-		0.22	4	-255.2	518.6	0.719	0.205
	3	-1.24	-			-	-		-	4	-255.6	519.4	1.520	0.138
GSAP	1	-0.29	X							3	-236.2	478.5	-	0.819
GTTA	1	-0.08	-			-		-		2	-120.2	244.5	-	0.314
	2	-0.08	-			-		0.23		3	-119.5	245.2	0.702	0.221
RUTUint	1	-1.09	X							3	-103.5	213.2	-	0.580
	2	-1.29	-							2	-104.9	213.8	0.646	0.420

Table S6 continued

Species	Model rank	Inter-cept	Geo-locator	Sex	Nest fate	Previously marked	Body mass	Day of capture	Marker mass	k	Dev-iance	AICc	ΔAICc	w_i
RUTUmor	1	-1.01	-	-		-				5	-73.5	157.4	-	0.436
	2	-1.30	X	-		-				6	-73.0	158.7	1.249	0.233
BLTU	1	0.11	-	-			-0.73	-	-	2	-49.0	102.2	-	0.107
	2	-1.12	-	X			-	-1.00	-	4	-47.0	102.6	0.418	0.087
	3	-0.47	-	X			-0.55	-	-	4	-47.1	102.6	0.460	0.085
	4	-0.95	-	X			-0.54	-0.97	-	5	-46.0	102.7	0.540	0.082
	5	-0.60	-	X			-	-	-	3	-48.2	102.8	0.619	0.079
	6	0.23	X	-			-0.75	-	-	3	-48.4	103.2	0.976	0.066
	7	0.11	-	-			-0.73	-	-0.25	3	-48.7	103.6	1.457	0.052
	8	0.11	-	-			-0.74	0.16	-	3	-48.9	104.1	1.875	0.042
REKNrog	1	0.27	-			-	0.79			2	-19.7	43.7	-	0.338
	2	0.25	-			-	-			1	-21.3	44.7	0.909	0.215
REKNruf	1	-0.99	-							1	-493.7	989.5	-	0.626
	2	-1.02	X							2	-493.2	990.5	1.032	0.374
AMGP	1	-1.23	-	-	-	-	-0.52	-0.98	-0.69	6	-87.3	187.1	-	0.068
	2	-1.24	-	-	-	-	-0.64	-0.91	-	5	-88.7	187.7	0.620	0.050
	3	-2.06	-	-	X	-	-	-1.05	-0.92	7	-86.5	187.7	0.627	0.050
	4	-1.20	-	-	-	-	-	-0.91	-0.85	5	-88.8	188.1	0.970	0.042
	5	-1.18	-	-	-	X	-0.57	-1.00	-0.70	7	-86.8	188.3	1.191	0.038
	6	-1.60	X	-	-	-	-0.68	-0.87	-	6	-87.9	188.4	1.261	0.036
	7	-1.95	-	-	X	-	-0.36	-1.06	-0.79	8	-85.8	188.6	1.505	0.032
	8	-1.50	X	-	-	-	-0.54	-0.94	-0.48	7	-86.9	188.6	1.532	0.032
GRSN	1	-0.61	-							2	-53.7	111.5	-	0.716
	2	-0.73	X							3	-53.5	113.4	1.854	0.284
GRKN	1	0.19	X							3	-91.7	189.5	-	0.726
	2	0.49	-							2	-93.7	191.4	1.952	0.274
BTGO	1	0.81	-	-						1	-25.5	53.0	-	0.477
	2	1.04	X	-						2	-25.2	54.5	1.517	0.223
WHIMhud	1	0.01	-	-	-					2	-36.1	76.3	-	0.280
	2	-0.21	X	X	-					3	-35.3	77.1	0.736	0.194
	3	-0.14	X	-	-					3	-35.6	77.6	1.277	0.148
WHIMisl	1	-1.03	X	X	-	-				3	-46.2	98.8	-	0.278
	2	-1.30	X	X	X	-				4	-45.4	99.3	0.486	0.218
	3	-0.23	-	-	-	-				2	-48.0	100.1	1.323	0.143
	4	-0.45	-	-	X	-				3	-47.2	100.8	1.967	0.104
FECU	1	-1.09	X							2	-16.2	36.9	-	0.847

Table S7. Top model sets for GLMMs testing sublethal effects of geolocators for five species of Arctic-breeding shorebirds pooled. Submodels with ΔAICc < 2.0 are shown here, and are ranked and sorted by descending model weight (wi). All submodels included random effects of species, site, and year. See Table S4 for definitions of abbreviations.

Response	Model rank	Intercept (control)	Geolocator	Nest fate	Sex	Previously marked	Date difference	Nest age difference	k	Deviance	AICc	ΔAICc	w_i
Interannual breeding movements	1	4.08	-	X	X	-			9	-1205.3	2428.9	-	0.397
	2	4.06	-	X	X	-0.15			10	-1204.5	2429.2	0.32	0.339
Proportional change in body mass	1	0.001	-				-	-	5	476.9	-943.6	-	0.288
	2	0.004	X				-	-	6	477.5	-942.9	0.71	0.202
	3	0.001	-				-0.005	-	6	477.1	-942.0	1.63	0.127
	4	0.001	-				-	0.003	6	476.9	-941.7	1.89	0.112

Table S8. Model-averaged effects of geolocator orientation on nest success and return rate for semipalmated sandpipers at site B03 and return rate for American golden-plovers at site B16. Effect size estimates are given as mean (SE), and effects of two geolocator leg attachments were tested: PAB = parallel-band; PEF = perpendicular-flag. Top model sets are provided in Table S9; sample sizes are in Table S2. See Table S4 for definitions of abbreviations.

		Intercept (control)	Geolocator			Nest fate			Previously marked		Day of capture	
			PAB	PEF		Hatched	Unknown					
Species	Response	Mean (SE)	Mean (SE)	Mean (SE)	RI	Mean (SE)	Mean (SE)	RI	Mean (SE)	RI	Mean (SE)	RI
SESA	Nest success	0.16 (0.29)	**0.01 (0.38)**	**-2.98 (1.04)**	**1.00**							
	Return rate	-0.89 (0.29)	**-1.31 (0.43)**	**-0.64 (0.48)**	**1.00**	**0.79 (0.30)**	**0.48 (0.35)**	**1.00**	0.34 (0.34)	0.35		
AMGP	Return rate	-1.63 (0.82)	1.49 (0.81)	1.30 (0.76)	0.39						**-0.98 (0.50)**	**0.82**

Table S9. Top model sets for covariates of nest fate and return rate for semipalmated sandpipers at site B03 and American golden-plovers at site B16 with either parallel-band or perpendicular-flag geolocator attachments. All submodels included random effects of year. See Table S4 for definitions of abbreviations.

Species	Response	Model rank	Intercept (control)	Geolocator type	Nest fate	Previously marked	Day of capture	k	Deviance	AICc	ΔAICc	w_i
SESA	Nest fate	1	0.16	X				4	59.2	126.8	-	0.996
		2	-0.20	-				2	-67.0	138.0	11.28	0.004
	Return rate	1	-0.87	X	X	-		6	-140.0	292.3	-	0.535
		2	-0.92	X	X	X		7	-139.5	293.5	1.22	0.291
AMGP	Return rate	1	-1.04	-			-0.71	3	-50.0	106.3	-	0.423
		2	-2.30	X			-1.29	5	-47.9	106.5	0.24	0.374
		3	-	-			-	2	-52.0	108.1	1.79	0.172

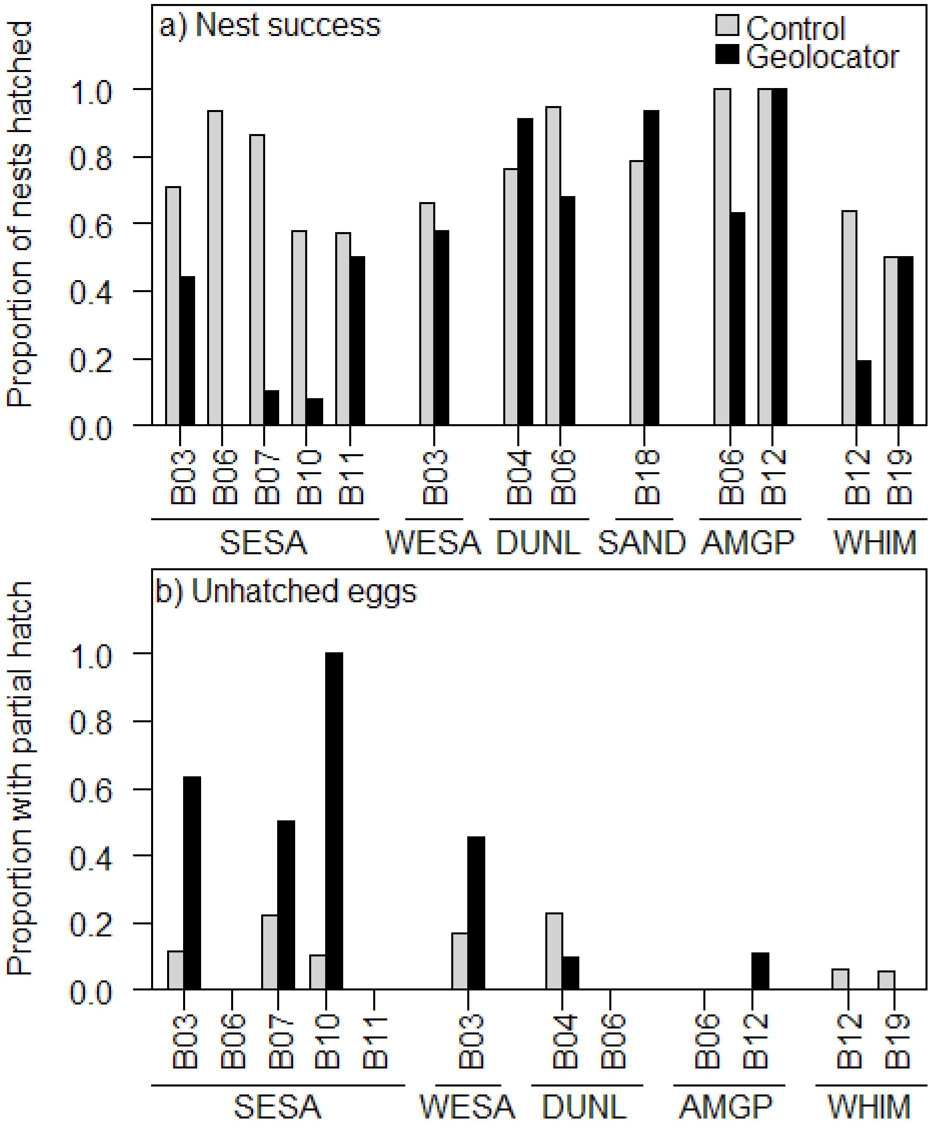

Figure S1. Nest success and partial hatching of clutches of control and geolocator nests for Arctic-breeding shorebirds. a) Proportion of nests hatched of those with known fate for control and geolocator groups of six species of Arctic-breeding shorebirds at a subset of sites. b) Proportion of hatched nests with unhatched eggs remaining for control and geolocator birds for five species. A dash instead of a bar indicates no data available for a given group to distinguish from groups for which no successful nests or no unhatched eggs were recorded. Species are ordered from smallest to largest; species codes are defined in Table 1 and site codes are in Table S1; sample sizes are given in Table S2.

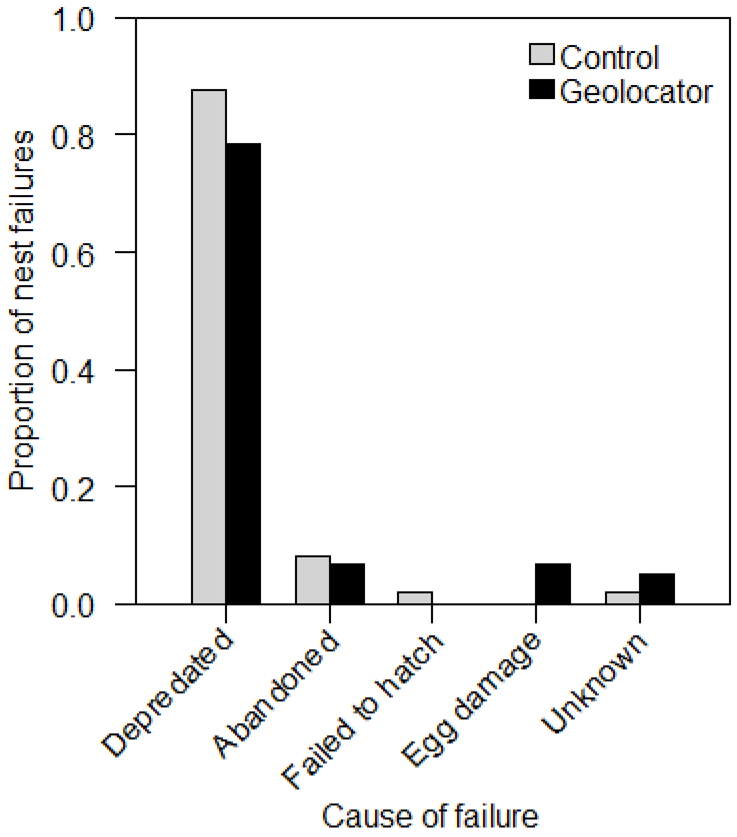

Figure S2. Cause of failure for control and geolocator semipalmated sandpiper nests that did not hatch, which together comprised 26% of nests with known fate.

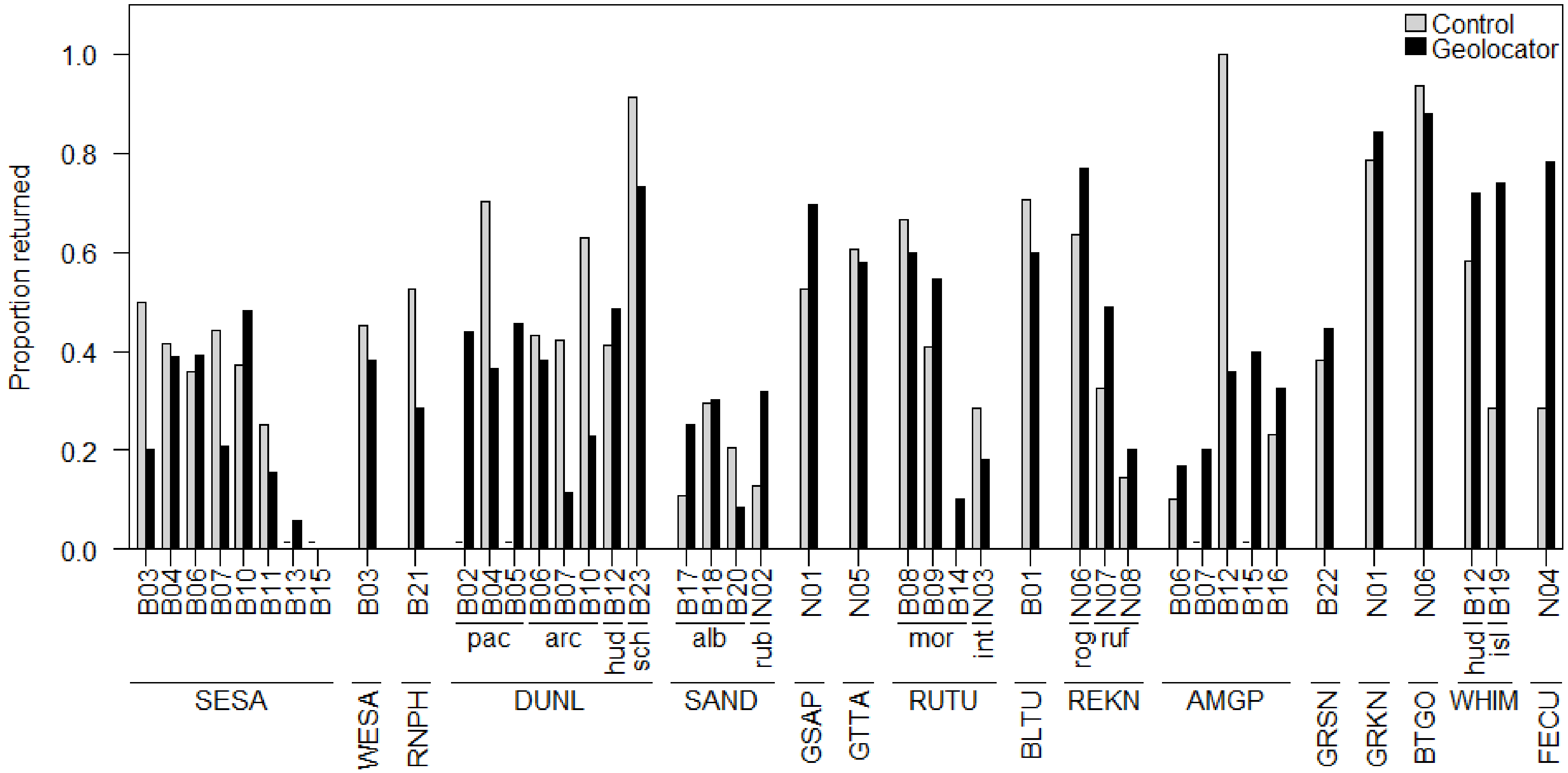

Figure S3. Proportion of shorebirds with and without geolocators that returned to each site one year after capture. A dash instead of a bar indicates no data for a given group to distinguish from cases where birds were marked but none returned. Species are ordered from smallest to largest; species codes are defined in Table 1, site codes are given in Table S1, and sample sizes are given in Table S2.
